# Tracking soil health and potentially toxic elements (PTEs) across land-use types using physico-chemical, magnetic, and geochemical proxies: a case study from Manipal, Southwestern India

**DOI:** 10.1007/s10653-025-02665-9

**Published:** 2025-08-19

**Authors:** Jai Vishnu Degvekar, Esha Ulhas Gadekar, O. Darshana, Jagath Chand, Vadakkeveedu Narayan Amrish, Jithin Jose, K. Priya, Santhosh Prabhu, Anish Kumar Warrier

**Affiliations:** 1 Department of Sciences, Manipal Institute of Technology, Manipal Academy of Higher Education, Manipal, 576104 Karnataka India; 2 Department of Civil Engineering, Manipal Institute of Technology, Manipal Academy of Higher Education, Manipal, 576104 Karnataka India; 3 Department of Marine Geology, Mangalore University, Mangalagangothri, 574199 Karnataka India; 4 Centre for Climate Studies, Department of Civil Engineering, Manipal Institute of Technology, Manipal Academy of Higher Education, Manipal, 576104 Karnataka India

**Keywords:** Soil quality, Environmental magnetism, Forest soil, Industrial soil, Pollution indices, Udupi district

## Abstract

This study investigates spatial variations in soil quality across five distinct land-use types—forest, roadside, agricultural, industrial, and residential—in the Manipal region of southern India. Ten surface soil samples from each land-use category were analysed for physico-chemical properties, geochemical composition, and environmental magnetic parameters to evaluate soil degradation and identify potential pollution sources. Marked variations were observed in electrical conductivity, pH, and salinity, with industrial and roadside soils exhibiting elevated values indicative of anthropogenic impact. Concentrations of potentially toxic elements (PTEs), including Pb, Cr, Cu, and Zn, were significantly higher in roadside and industrial areas, frequently exceeding recommended safety thresholds. Environmental magnetic measurements, such as low-frequency magnetic susceptibility (χ_lf_) and frequency-dependent susceptibility (χ_fd_%), effectively differentiated between anthropogenic and pedogenic magnetic inputs. Principal Component Analysis (PCA) highlighted distinct contamination patterns and revealed strong correlations between magnetic parameters and PTE concentrations. Pollution indices—including the Pollution Load Index (PLI), Enrichment Factor (EF), and Contamination Factor (CF)—identified notable contamination in industrial soils (PLI > 1.3). Overall, the findings underscore increasing environmental pressures on soil systems in rapidly urbanizing regions and demonstrate the value of environmental magnetism as a cost-effective, non-destructive tool for early detection and long-term monitoring of soil pollution. These insights can inform sustainable land management strategies and guide policy frameworks aimed at safeguarding soil health and ecological resilience.

**Supplementary Information:**

The online version contains supplementary material available at 10.1007/s10653-025-02665-9.

## Introduction

Soil is a fundamental component of terrestrial ecosystems, crucial for sustaining plant growth, regulating water, cycling nutrients, and supporting biodiversity (Krishna & Govil, 2007; Simonson, 1968). However, increasing anthropogenic pressure has led to widespread soil contamination, posing risks to food security, human health, and ecosystem functions (Silva et al., 2021; Rahmani et al., 2022). Soils, due to their retention capacity, serve as effective media for long-term environmental monitoring, particularly for assessing the accumulation of potentially toxic elements (PTEs) such as Pb, Cd, and Zn (Krishna & Govil, 2005; Soodmand et al., 2025; Soupios et al., 2016).

PTEs, whether of geogenic or anthropogenic origin, interfere with soil fertility and microbial activity, disrupting key biogeochemical processes such as mineralization, nitrification, and nutrient availability (Mazur et al., 2015; Wyszkowski & Brodowska, 2020). The natural geochemical cycles that regulate soil fertility and nutrient availability are especially vulnerable to disruption from trace elements introduced through fertilizers, sewage sludge, and atmospheric deposition ( , 1998; Mazur et al., 2015; Wyszkowski & Brodowska, 2020). Elements such as cadmium (Cd), lead (Pb), and zinc (Zn) can inhibit soil microbial communities, impairing vital functions like nitrogen mineralization and nitrification. These disruptions reduce soil fertility, crop productivity, and nutrient use efficiency. Moreover, the long-term accumulation of trace metals alters redox reactions, metal–organic complexation, and organic matter decomposition, ultimately increasing the mobility of both nutrients and pollutants in agricultural soils (Nziguheba & Smolders, 2008; Plante, 2007; Singh, 1994).

Such changes in soil chemistry and biological functioning are compounded by external sources including industrial discharges, vehicular emissions, fertilizers, pesticides, and urban waste (Ali et al., 2019; Golia et al., 2008a). Particularly in urban and peri-urban areas, road dust, domestic waste, and industrial runoff elevate metal concentrations beyond natural background levels (Al-Khashman, 2004; Fergusson & Kim, 1991).

In this context, environmental magnetism offers a promising, rapid, and cost-effective approach for assessing soil contamination. Magnetic parameters, especially low-frequency magnetic susceptibility (χ_lf_), show strong correlations with PTE concentrations in polluted environments such as roadside verges and industrial zones (Dankoub et al., 2012; Jordanova et al., 2008; Warrier et al., 2014). Since magnetic minerals often co-occur with PTEs in fine-grained soil fractions, these proxies can effectively reflect both the sources and intensities of contamination (Hays et al., 2011; Lu et al., 2008).

Given these concerns, Manipal—a rapidly urbanizing township in Udupi district, Karnataka, India—offers a unique setting to investigate soil health across diverse land-use types. The region supports over 35,000 transient residents annually and includes agricultural lands, industrial zones, and fragmented forest patches within a compact area (Government of Karnataka, 2023–2024). Although earlier studies have characterized the mineralogical and geochemical properties of lateritic soils in the region (Budihal & Pujar, 2018; Nayak et al., 2023), a comprehensive assessment incorporating PTEs contamination and magnetic indicators of soil health across land-use gradients is still lacking.

This study addresses that gap by investigating five representative land-use types—forested, roadside, agricultural, industrial, and residential—within a 5 km radius in Manipal. It applies a multi-proxy framework combining physico-chemical, geochemical, and environmental magnetic parameters to: (i) quantify PTE concentrations and their variation across land-use types, (ii) evaluate the suitability of magnetic proxies as indicators of soil contamination, and (iii) provide baseline data to support sustainable land management in tropical lateritic terrains.

The outcomes will inform regional policy, complement India’s Soil Health Card Mission, and contribute to Sustainable Development Goals (SDGs 3, 11, and 15). This integrated approach enhances current soil monitoring frameworks by demonstrating the potential of low-cost magnetic tools to track PTEs-related soil health degradation in rapidly developing landscapes.

## Study area

The present study was conducted in Manipal town, located in the Udupi district of Karnataka, India. Manipal lies approximately 5.2 km from the Udupi bus stand, at 13.3524°N latitude and 74.7868°E longitude, with an elevation of 73 m above mean sea level. It is situated between two major physiographic features: the Arabian Sea to the west and the Western Ghats to the east. Manipal experiences a tropical monsoon climate characterized by high annual rainfall, averaging 4338 mm (Pai et al., 2014) and a mean annual temperature of 26.7 °C (Jayashree et al., 2023; Kumar et al., 2017). The region is influenced by the southwest monsoon, with most precipitation occurring between June and September.

The central zone of Manipal is dominated by a world-class multidisciplinary university, along with associated urban settlements and small industrial estates. In contrast, the peripheral areas are marked by agricultural lands and scattered forest patches. The primary crops cultivated in these agricultural zones include arecanut, paddy, and seasonal vegetables (Government of Karnataka, Udupi District, 2023–2024).

The region’s topsoil is predominantly lateritic, characterized by its distinctive red colour and high iron oxide content, typical of tropical regions in southern India (Sandesh et al., 2022). These lateritic soils are the weathering products of gneissic and granitic rocks, and are known for their porous texture, low fertility, and acid nature. Soils in the Udupi district are broadly classified into red lateritic, yellow loamy, and sandy types, each varying in texture and mineral composition depending on topography and parent material. Geologically, the area is part of the Peninsular Gneissic Complex, which comprises granites, migmatites, and gneisses, representing some of the oldest crystalline formations in the Indian subcontinent (Radhakrishna & Vaidyanadhan, 1994). These are overlain by younger lithological units such as Kanara granite, biotite gneiss, and quartz-feldspathic gneiss. Extensive magmatization and metamorphic processes have contributed to the formation of varied rock types, including granite gneiss, migmatitic gneiss, and hornblende-biotite gneiss (Geological Survey of India, 1994).

## Materials and methodology

### Sampling

**Fig. 1:**
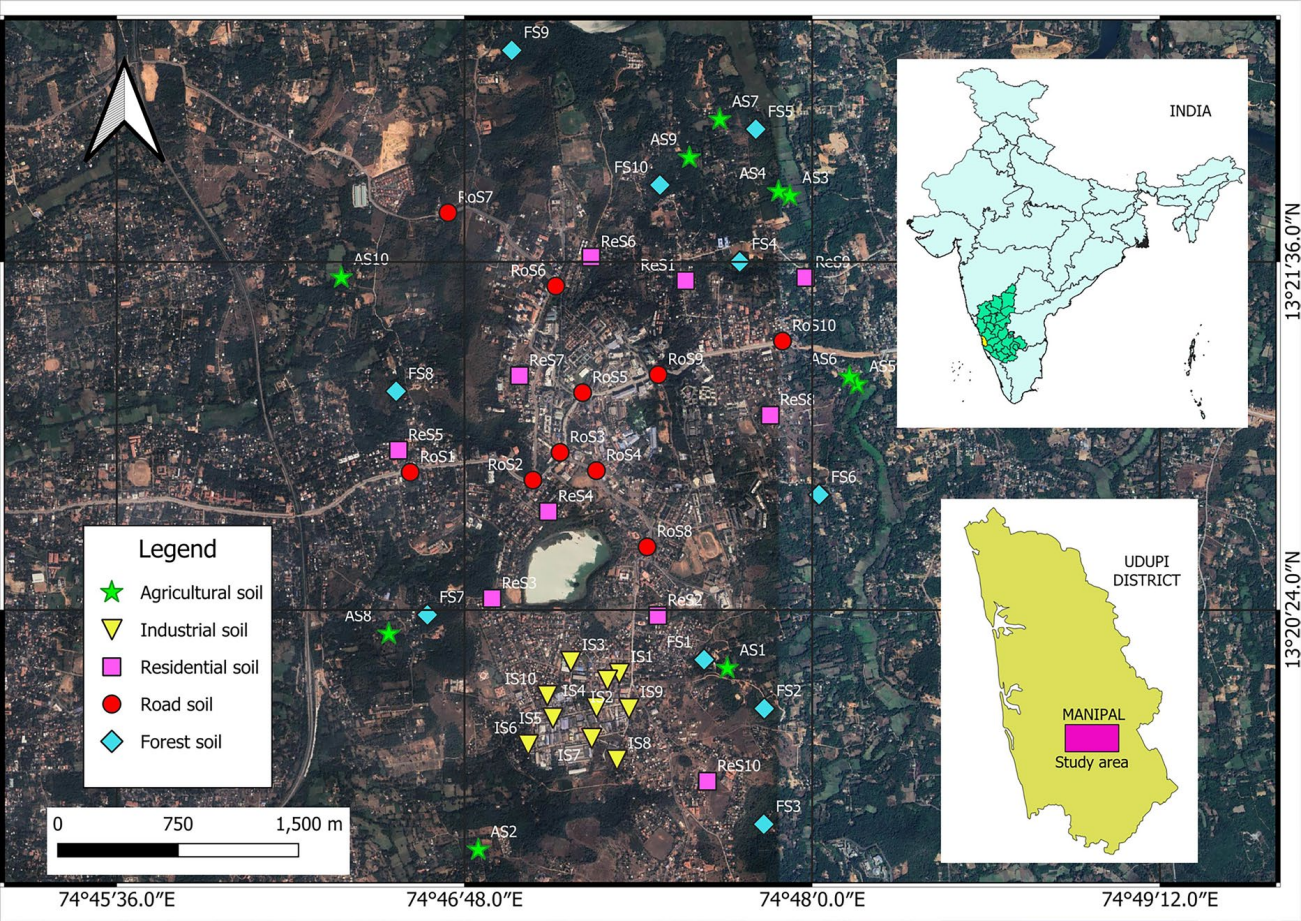
Satellite map of the study area in Manipal town, Udupi district, showing the locations of sampled sites across five land-use categories: forest soil, road soil, agricultural soil, industrial soil, and residential soil. The base map is derived from Google Earth imagery

A total of 50 surface soil samples were collected from a depth of 0–5 cm in and around the Manipal area, covering five distinct land-use types: industrial areas, roadsides, residential areas, agricultural lands, and forested areas, with 10 samples collected from each category as represented in Fig. 1 (Geographical coordinates provided in Supplementary Table S1). Approximately 1 kg of soil per sample was collected by marking a 50 × 50 cm grid on the ground. The soil within each grid was thoroughly mixed using a wooden spoon and stored in plastic Ziplock bags to avoid metallic contamination (Pathak et al., 2015). Roadside soil samples were specifically collected with a wooden spatula to prevent metal contamination, targeting areas near road dividers and junctions at the centre of the roads. Ten samples from each land-use category were collected to ensure adequate spatial representation, considering the study’s objectives to assess spatial variability, ensure accessibility across diverse terrains, and generate reliable baseline data on soil health for future environmental monitoring in the Manipal region.

### Physico-chemical analysis

On-site analysis of physico-chemical parameters was performed by preparing a 1:3 (soil:Milli-Q water) suspension in 50 mL borosilicate beakers. Parameters such as temperature, pH, salinity, and electrical conductivity were measured using a Hach HQ40d dual input multiparameter meter (Loska et al., 2004; Thunjai et al., 2001).

### Soil Colour determination

Soil colour was assessed at the sampling site using the Munsell Soil Colour Chart (MSCC). The closest match was identified by visually comparing soil samples to standardized colour chips, and corresponding hue, value, and chroma notations were recorded (Fan et al., 2017; Nodi et al., 2025). MSCC categorizes colour based on hue (shade), value (lightness), and chroma (intensity) (Łachacz & Załuski, 2023; Pegalajar et al., 2020).

### Geochemical analysis (ICP-OES)

The chemical composition of major and minor elements in all 50 samples was analysed using Inductively Coupled Plasma Optical Emission Spectroscopy (ICP-OES). Samples were air-dried, oven-dried at 40 °C, sieved to < 63 μm, and finely ground using an agate mortar and pestle. A 200 mg aliquot was treated with 3 mL of hydrogen peroxide (H_2_O_2_) to remove organic matter. Digestion was done using a tri-acid mixture (HNO_2_: HCl: HF = 5:3:2), and the resulting crystalline paste was diluted with 0.2 N HNO_2_ and filtered through 20 μm syringe filters. Fifteen milliliters of each digested sample solution were analyzed using Inductively Coupled Plasma Optical Emission Spectroscopy (ICP-OES) following the protocol outlined by Pathak et al. (2015). Certified Reference Materials (CRM), ERM-CC141 (standard loam soil), and procedural blanks were included in each batch as part of the quality control process. To assess analytical accuracy and precision, the CRM was digested alongside the soil samples using the same tri-acid digestion procedure (Hu & Qi, 2014). The accuracy and precision for all analyzed elements were within ± 5%, based on comparisons between CRM values and duplicate sample measurements.

### Environmental magnetic measurements

Dried samples were sieved (< 2 mm), packed into non-magnetic plastic vials, and measured using a Bartington MS2B dual-frequency susceptibility meter at 0.47 kHz and 4.7 kHz. Mass-specific magnetic susceptibility (χ) was calculated by normalizing κ to sample density. Calibration was done using a 1% Fe_2_O_4_ standard, and drift was checked after every ten samples (Joju et al., 2023; Reethu et al., 2023). Low-frequency susceptibility (χ_lf_) indicates the bulk ferrimagnetic content, while frequency-dependent susceptibility (χ_fd_) and χ_fd_% were calculated as: χ_fd_ = χ_lf_ – χ_hf_; χ_fd_% = ((χ_lf_ − χ_hf_)/χ_lf_) × 100 (Dearing et al., 1996; Thompson & Oldfield, 1986).

The remanence measurements were carried out at the Environmental Magnetism Laboratory, Department of Marine Geology, Mangalore University. Anhysteretic remanent magnetization (ARM) was induced using a Molspin AF demagnetizer (100 mT alternating field, 0.04 mT DC bias). The χ_ARM_ was calculated by dividing mass-specific ARM by the biasing field (31.84 Am^−1^) (Walden, 1999). Isothermal remanent magnetization (IRM) was induced at various field strengths (20 to 1000 mT) using a Molspin pulse magnetizer. Saturation IRM (SIRM) was obtained at 1 T. All remanence values were measured with a Molspin fluxgate spinner magnetometer. Magnetic mineralogy and grain size were inferred from χ_ARM_/χ_lf_, χ_ARM_/SIRM, SIRM/χ_lf_, and S-ratio indices (Table 1; Maher, 1988; Oldfield, 1991; Walden, 1999).

**Table 1:** Environmental magnetic measurements with their units, interpretation and instruments (modified after Maher, 1988; Thompson & Oldfield, 1986)

Magnetic measurements	Units	Interpretation	Instruments used
Low- and high- frequency susceptibility (χ_lf_ and χ_hf_)	10^−8^ m^3^ kg^−1^	Proportional to the concentration of magnetic minerals	Bartington susceptibility meter with a dual-frequency sensor
Frequency-dependent susceptibility (χ_fd_)	10^−8^ m^3^ kg^−1^	Proportional to the concentration of superparamagnetic grains	Bartington susceptibility meter with a dual-frequency sensor
Susceptibility of Anhysteretic Remanent Magnetization χ_ARM_	10^−5^ m^3^ kg^−1^	Proportional to the concentration of magnetic minerals of stable single domain size range	AF-demagnetiser with an ARM attachment and fluxgate magnetometer
Isothermal Remanent Magnetisation (IRM) and Saturation Isothermal Remanent Magnetisation (SIRM)	10^−5^ Am^2^ kg^−1^	Proportional to the concentration of remanence-carrying magnetic minerals	Pulse magnetizer and fluxgate magnetometer
χ_ARM_χ_lf_		Indicative of magnetic grain size. A higher (lower) ratio indicates a finer (coarser) magnetic grain size. Also indicates the presence of bacterial magnetite if the ratio is > 40	
χ_ARM_/χ_fd_		Indicative of magnetic grain size. A higher (lower) ratio indicates a finer (coarser) magnetic grain size. Also indicates the presence of bacterial magnetite if the ratio is > 1000	
χ_ARM_/SIRM	10^−5^ mA^−1^	Indicative of magnetic grain size. A higher (lower) ratio suggests a finer (coarser) magnetic grain size	
SIRM/χ_lf_	10^3^ Am^−1^	Indicative of magnetic grain size if the mineralogy is uniform. A higher (lower) ratio suggests a coarser (finer) grain size. Also indicates the presence of greigite if the ratio is > 30	
S-ratio = IRM_300mT_/SIRM		Relative proportions of ferrimagnetic and anti-ferromagnetic minerals (high ratio = a relatively high proportion of ferrimagnets)	
HIRM = SIRM—IRM_300mT_	10^−5^ Am^2^ kg^−1^	Proportional to the concentration of antiferromagnetic minerals like hematite, goethite etc	

### Statistical and risk assessment analysis

Descriptive statistics, including mean, standard deviation (SD), coefficient of variation (CV), and range, were computed to evaluate the distribution of metals (Mouli et al., 2006; Pathak et al., 2015). Principal Component Analysis (PCA) was conducted on the standardized dataset using PAST software, version 5.2.2, which is widely used for statistical analysis in scientific research (Hammer et al., 2001). The suitability of the dataset for PCA was assessed using Bartlett’s test of sphericity and the Kaiser–Meyer–Olkin (KMO) measure of sampling adequacy. To assess the level of metal contamination in surface and subsurface soils, geo-accumulation index (I_geo_), contamination factor (CF), and pollution load index (PLI) were calculated (Supplementary Table S2). The mean values of the ten forest soil samples were used as a geochemical baseline due to their minimal anthropogenic influence.

#### Contamination factor (CF)

Contamination factor was used to predict the degree of metal pollution in the area (Caeiro et al., 2005; Hakanson, 1980; Pathak et al., 2015). The CF is mathematically expressed as:

$$CF=\frac{{C}_{sample}}{{C}_{LSS}}$$

where C_sample_ denotes the metal concentration present in collected soil samples and C_LSS_ denotes the concentration of background soil values. The low contamination is denoted by CF < 1, moderate contamination by 1 ≤ CF < 3, significant contamination by 3 ≤ CF ≤ 6, and very high contamination by CF > 6 (Supplementary Table S2).

#### Pollution load index (PLI)

Pollution Load Index (Djordjević et al., 2012; Joju et al., 2024; Tomlinson et al., 1980) which is a composite index based on all elements, allows the assessment of the overall element pollution. It is mathematically expressed as:

$$PLI=({{CF}_{1}\times {CF}_{2}\times {CF}_{3}\dots \times {CF}_{n})}^{1/n}$$

where n is the total number of metals and CF is the contamination factor of each element as calculated above. A value of one is considered as the baseline level of pollution and a value of greater than one would indicate decrease in sampling site quality (Tomlinson et al., 1980).

#### Geoaccumulation index (I_geo_)

The level of toxic metal accumulation in sediments is measured by the Geoaccumulation Index (I_geo_) (Barbieri, 2016; Joju et al., 2024). The geoaccumulation index is given by the formula:

$${I}_{geo}={log}_{2}({C}_{n}/1.5{B}_{n})$$

where C_n_ represents the concentrations of each element and B_n_ represents the chosen background soil values. 1.5 is used as a correction factor for inherent natural lithogenic variations in metal concentrations (Joju et al., 2024; Nowrouzi & Pourkhabbaz, 2014).

#### Enrichment factor

The Enrichment Factor (EF) is a commonly used index to differentiate between natural and anthropogenic sources of metal contamination in soils. It is determined by comparing the ratio of a metal in the sample with a background material to a stable reference element, like Al or Fe (Loska et al., 2004) given by the formula:

$$EF= \frac{{c}_{n(sample)}/{Al}_{(sample)}}{{B}_{n(background)}/{Al}_{(background)}}$$

where the content of the examined element in the examined environment is denoted by C_n(sample)_, the content of the reference element in the examined environment by C_ref(sample)_, the content of the examined element in the reference environment by B_n(background)_, and the content of the reference element in the reference environment by B_ref(background)_. EF < 2 values indicate a crustal origin, EF > 2 indicate anthropogenic origin and greater than 10 indicates purely anthropogenic origin (Nowrouzi & Pourkhabbaz, 2014; Rubio et al., 2000).

## Results and discussions

### Physico-chemical parameters

In the Manipal region, physico-chemical analysis of soils across different land-use types—forest, roadside, agricultural, industrial, and residential—revealed significant differences in pH, salinity, and electrical conductivity (EC). These parameters, influenced by both natural and anthropogenic activities, are critical indicators of soil health (Golia et al., 2008a; Krishna & Govil, 2007). The Munsell soil colour index corresponding to each sample is presented in Supplementary Table S3.

**Fig. 2:**
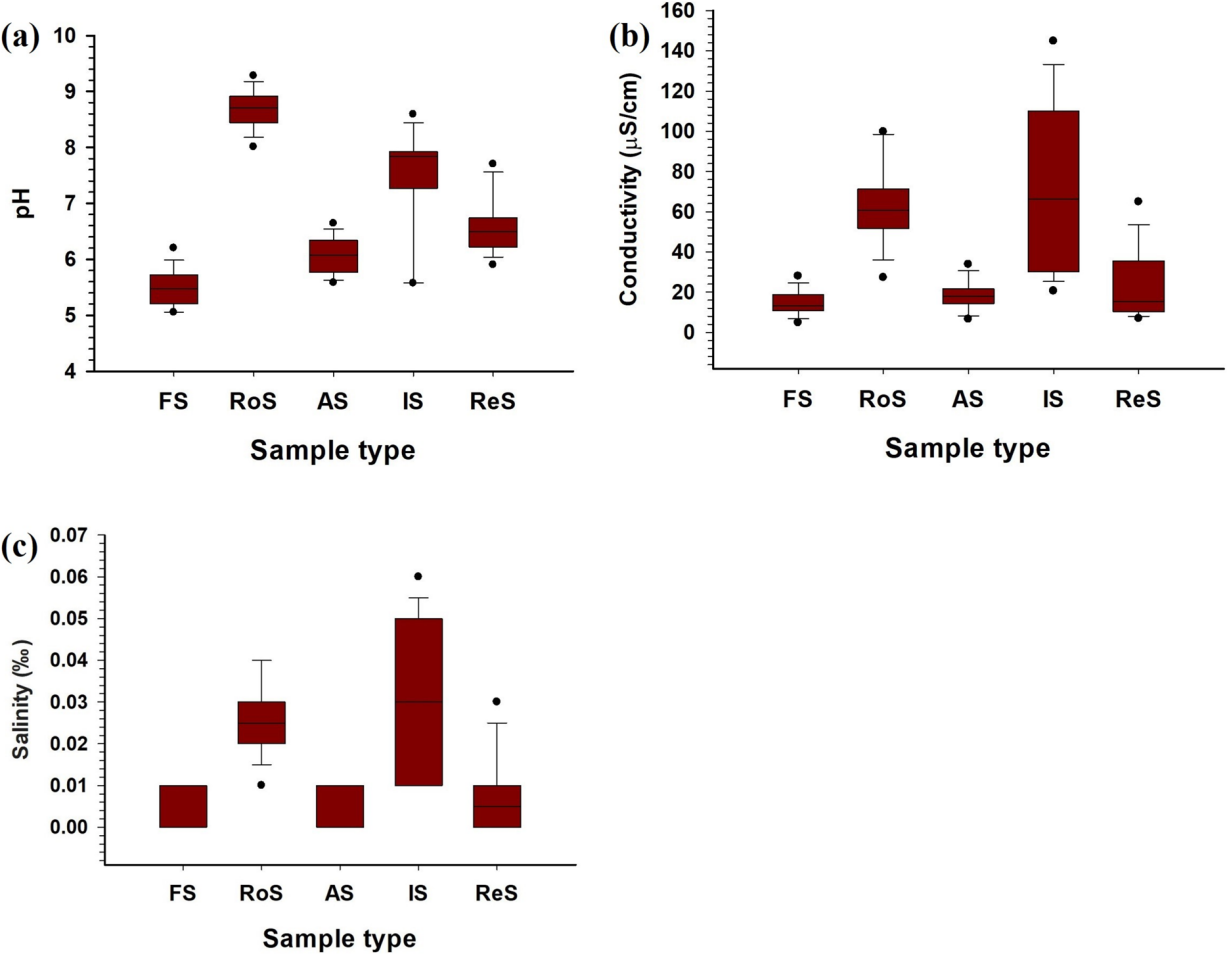
Box plots showing the distribution of physico-chemical parameters across the different soil types: (**a**) pH, (**b**) electrical conductivity (µS/cm), and (**c**) salinity (‰). The boxes represent interquartile ranges, the lines within boxes indicate the median, and the dots represent outliers

Forest soils had an average pH of 5.5 (± 0.35) (Supplementary Table S4; Fig. 2), with the lowest value (5.0) recorded at FS3 near Manchi Waterfall (Fig. 1), indicating acidic conditions. This is attributed to the thick organic cover and microbial decomposition releasing organic acids (Sandesh et al., 2022). Additionally, heavy rainfall in the region enhances leaching of acidic parent materials, contributing further to acidity (Golia et al., 2008a; Lingappa et al., 2024). These trends are consistent with studies from the Western Ghats and coastal Karnataka, where lateritic soils under intense rainfall exhibit acidic pH (Kumar et al., 2017). EC values were low (mean: 14.78 µS/cm), indicating minimal ionic concentrations and pristine conditions with limited human impact (Golia et al., 2008b). Salinity was very low (mean: 0.003‰), supporting previous observations (Sandesh et al., 2022).

Roadside soils showed the highest pH value of 9.28 at RoS10 (Fig. 1), with a mean of 8.6 (± 0.36), suggesting alkaline conditions (Supplementary Table S4; Fig. 2). This may be due to alkaline dust deposition from vehicular emissions, cement particles, and construction debris—similar to findings in urban environments like Surat and Mumbai (Al-Khashman, 2004; Krishna & Govil, 2005). Mean EC was 63.33 µS/cm (± 22.32), reflecting the accumulation of soluble salts, PTEs, and pollutants introduced via emissions and runoff. However, salinity remained low (0.026‰).

Agricultural soils were sampled from arecanut plantations and paddy fields, representing contrasting water management regimes. The mean pH was 6.05 (± 0.34) indicating slightly acidic conditions optimal for most crops in Karnataka (Sujatha et al., 2017). These values align with findings from Udupi and Dakshina Kannada, where nitrogen-based fertilizers contribute to gradual acidification (Choudhary & Kharche, 2018; Golia et al., 2008a). Paddy fields had higher EC (21.42 µS/cm) than arecanut soils (12.3 µS/cm), likely due to waterlogging and fertilizer accumulation (Sujatha et al., 2017). Arecanut soils, with better drainage, showed lower EC due to salt leaching (Bhat & Sujatha, 2009). Salinity was negligible (0.007‰), well below thresholds for harmful effects (Rengasamy, 2010; Rengasamy et al., 2003).

Industrial soils had a mean pH of 7.44 (± 1.04), ranging from neutral to slightly alkaline, typical of areas influenced by construction material and industrial effluents (Govil et al., 2008). The highest EC (144.7 µS/cm) value across all land-use types was recorded at IS10 near a concrete plant, indicating high ionic concentration due to metal deposition and waste discharge. These trends mirror findings from Surat and Thane-Belapur industrial areas (Krishna & Govil, 2007; Pathak et al., 2015). Salinity remained relatively low (mean: 0.031‰ ± 0.02).

Residential soils exhibited a mean pH of 6.62 (± 0.55) (Supplementary Table S4), reflecting mildly acidic to neutral conditions. Soil chemistry here appears influenced by domestic waste disposal, irrigation, and leaching from construction materials. EC averaged 23.87 µS/cm (± 18.62), suggesting inputs from greywater, sewage leakage, and organic waste, consistent with other urban studies (Xie et al., 2019). Salinity was low (0.008‰), possibly due to regular irrigation of lawns and gardens, consistent with values reported by Wagh et al. (2013), though lower than salinity levels noted by Lyu and Chen (2016; 0.086‰) in urban soils.

### Environmental magnetic properties of soils

**Fig. 3:**
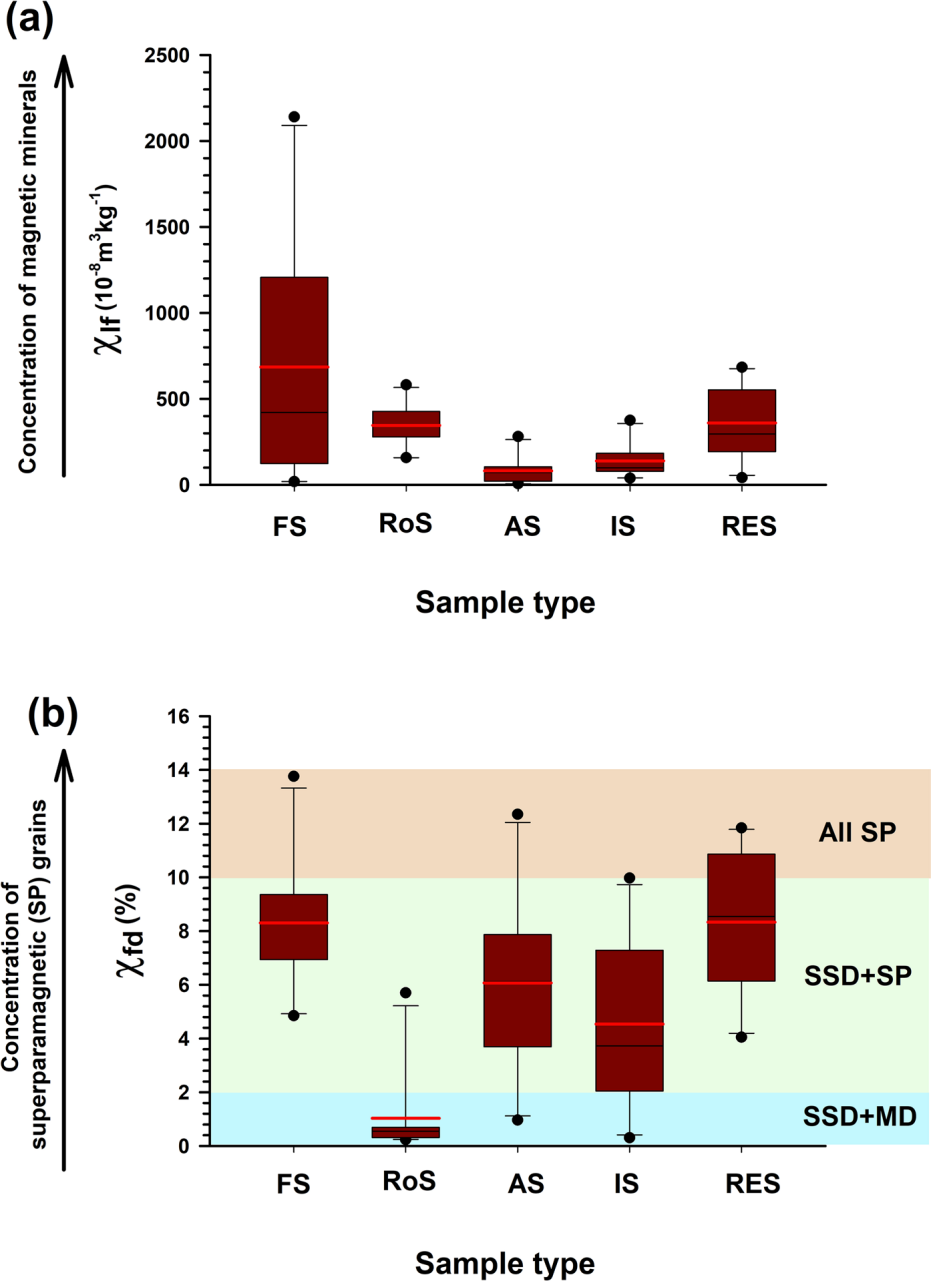
Box plots showing variations in magnetic parameters across different land-use types: (**a**) low-frequency magnetic susceptibility (χ_lf_) indicating the concentration of magnetic minerals, and (**b**) frequency-dependent susceptibility (χ_fd_%) reflecting the concentration of superparamagnetic (SP) grains. Shaded zones in (**b**) represent magnetic domain interpretations: stable single domain + multi-domain (SSD + MD), stable single domain + superparamagnetic (SSD + SP), and dominantly superparamagnetic (All SP)

Magnetic parameters such as low-frequency magnetic susceptibility (χ_lf_) and frequency-dependent susceptibility (χ_fd_%) are reliable indicators of magnetic mineral concentrations in soils and serve as valuable proxies for distinguishing pedogenic versus anthropogenic sources (Amrutha et al., 2021; Dearing et al., 1996). χ_lf_ reflects the abundance of ferrimagnetic minerals, predominantly magnetite and maghemite (Amrutha et al., 2021). Figure 3a presents boxplots of χ_lf_ values across the studied land-use types.

Forest soils exhibited the highest χ_lf_, averaging 685.09 × 10^−8^ m^3^ kg^−1^ (range: 19.1–2140 × 10^−8^ m^3^ kg^−1^; Supplementary Table S5), consistent with observations from undisturbed forested terrains (Cao et al., 2021; Declercq et al., 2019). This is attributed to abundant pedogenic ferrimagnetic minerals formed through organic matter decomposition and microbial processes (Amrutha et al., 2021; Dekkers, 2004). Humic acids released during decomposition aid iron mobilization, promoting fine-grained magnetic oxide formation (Declercq et al., 2019).

**Fig. 4:**
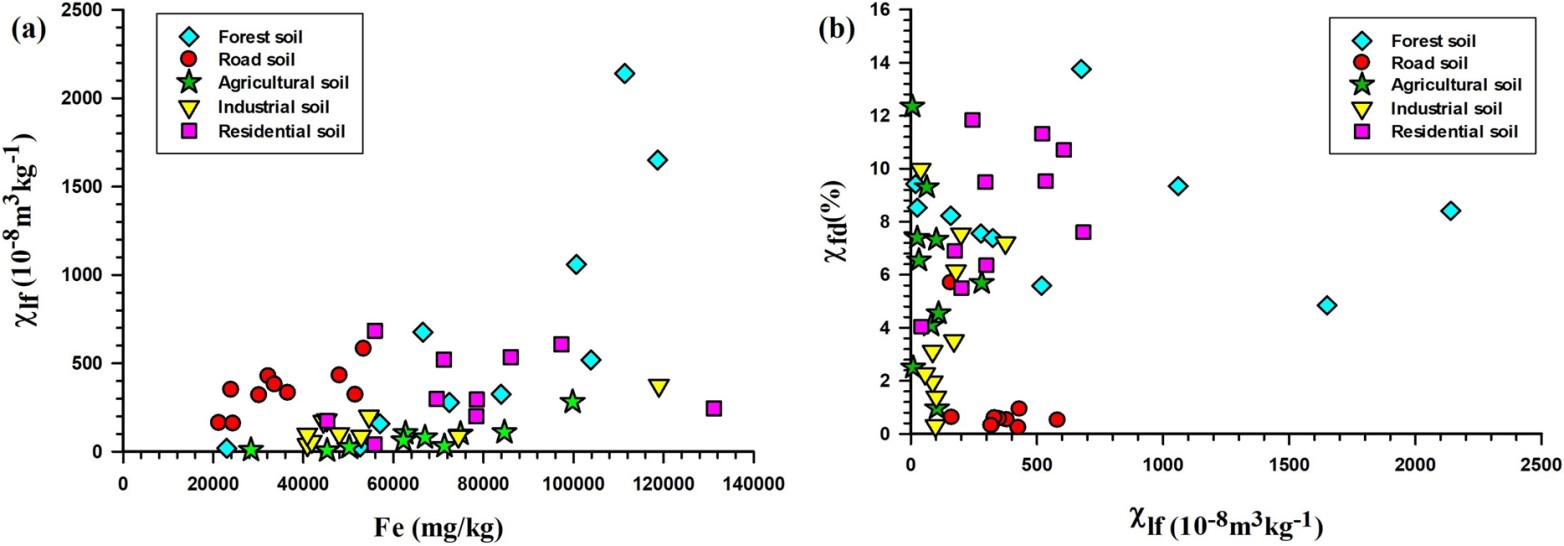
Scatter plots showing magnetic and geochemical relationships across different land-use types: (**a**) correlation between iron (Fe) concentration (mg/kg) and low-frequency magnetic susceptibility (χ_lf_, 10^−8^ m^3^kg^−1^), and (**b**) relationship between χ_lf_ and frequency-dependent susceptibility (χ_fd_%, an indicator of superparamagnetic grain content)

**Fig. 5:**
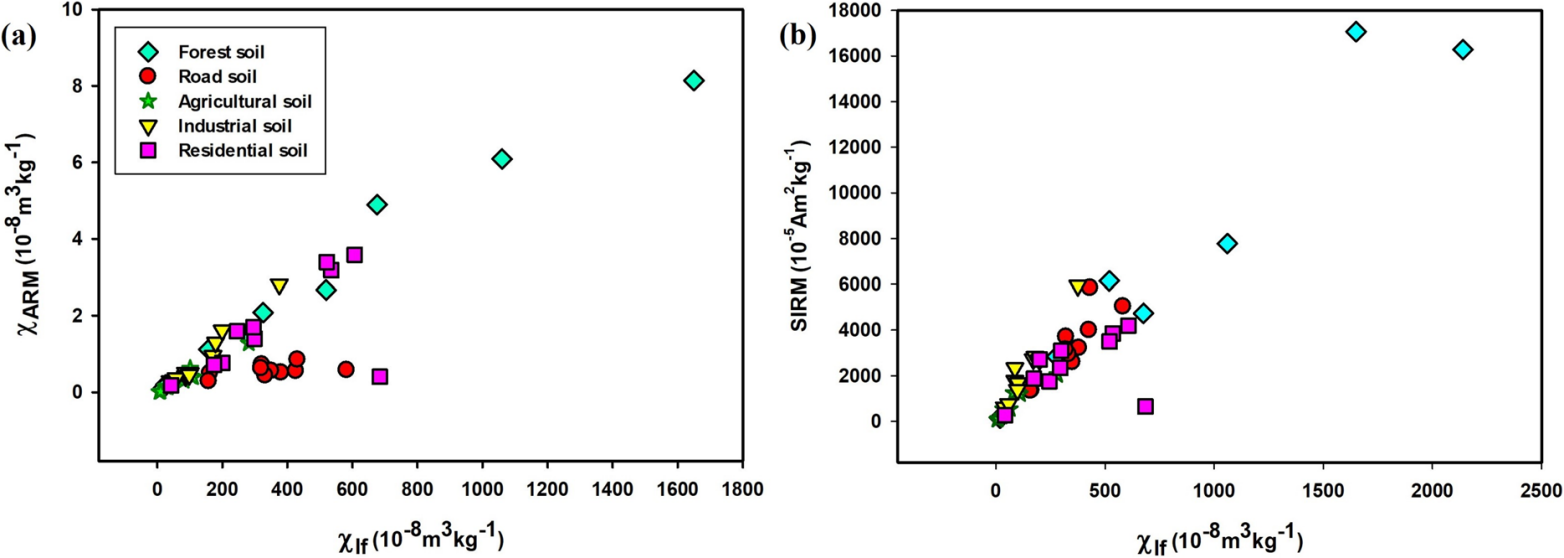
Scatter plots showing relationships between magnetic parameters across different land-use types: (**a**) χ_lf_ *vs.* χ_ARM_, highlighting the concentration of magnetic minerals associated with stable single-domain (SSD) grains; and (**b**) χ_lf_ *vs.* SIRM, reflecting the overall concentration of magnetic minerals in the soil samples

**Fig. 6:**
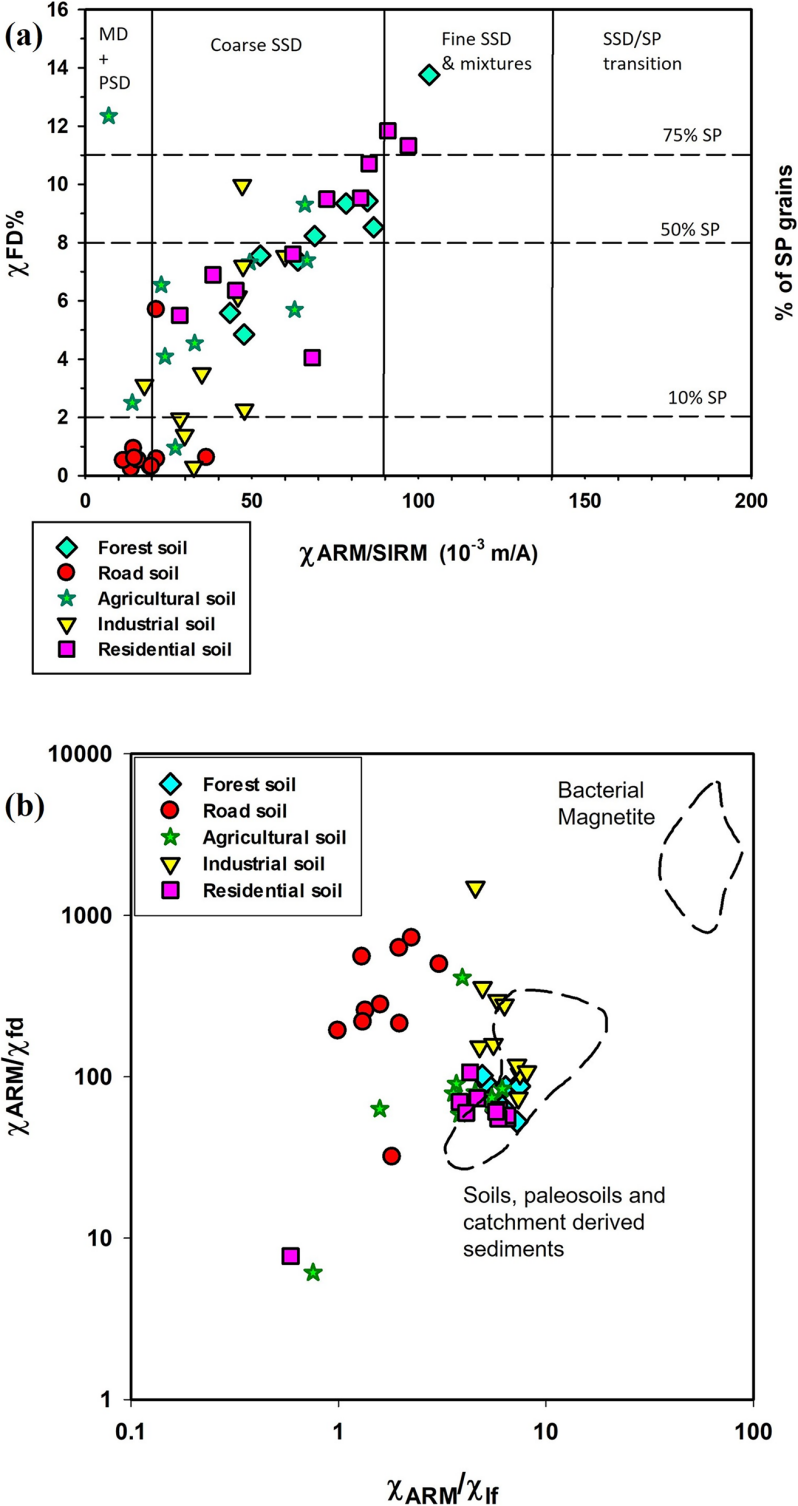
Biplots illustrating magnetic grain size and domain state interpretations for soil samples from different land-use types: (**a**) χ_ARM_/SIRM *vs.* χ_fd_%, used to differentiate magnetic domain states—multi-domain (MD), pseudo-single domain (PSD), single domain (SSD), and superparamagnetic (SP)—and to infer the relative abundance of SP grains; and (**b**) χ_ARM_/χ_lf_ *vs.* χ_ARM_/χ_fd_, used to distinguish between soil-derived magnetic minerals and biogenic (bacterial) magnetite. The observed values of χ_ARM_/χ_lf_ (< 40) and χ_ARM_/χ_fd_ (< 1000) suggest that the magnetic minerals in the study area are not influenced by bacterial magnetite

The χ_fd_% of forest soils (4.8–13.75%; average 8.30%) indicates SP and SSD grain dominance (Fig. 3b), supported by a χ_ARM_ average of 14.27 × 10^−5^ m^3^ kg^−1^ (Figs. 4 and 5). Strong correlations—χlf-χARM (r = 0.75; p <0.0012; n = 10; Fig. 5a), χ_lf_-SIRM (r = 0.97; p < 0.0001; n = 10; Fig. 5b), and χ_lf_-IRM_300mT_ (r = 0.97; p < 0.0001; n = 10)_lf_-_ARM_—underscore the control of fine ferrimagnetic mineral over magnetic properties (Supplementary Table S6). Biplots of χ_fd_% *vs.* χ_ARM_/SIRM and χ_ARM_/χ_lf_ *vs.* χ_ARM_/χ_fd_ (Fig. 6a, b) confirm dominance of SP + SSD grains. S-ratio values (~ 0.94) and moderate HIRM (~ 139.5 × 10^−5^ A m^2^ kg^−1^) further suggest magnetite dominance with minor hematite/goethite presence.

Forest soils from the study area exhibited higher values of χ_fd_% (average: 8.30%) and χ_ARM_ (average: 14.27 × 10^−5^ m^3^ kg^−1^), indicating a substantial presence of fine pedogenic magnetic minerals likely formed through enhanced organic activity. In contrast, Ananthapadmanabha et al. (2014) reported moderate χ_lf_ (average: 581.5 × 10^−8^ m^3^ kg^−1^) and low χ_fd_% (average: 2.2%) in topsoil (0–10.5 cm), suggesting a predominance of coarser, lithogenic magnetic grains. Similarly, pristine soils examined by Amrutha et al. (2021) demonstrated a wide range of χ_lf_ values (58.17–6249.57 × 10^−8^ m^3^ kg^−1^; average: 881.10 × 10^−8^), reflecting mixed lithogenic and pedogenic contributions.

Roadside soils exhibited moderate χ_lf_ values, averaging 346.1 × 10^−8^ m^3^ kg^−1^, with peak concentrations recorded near high-traffic junctions (Fig. 1), aligning with earlier findings (Lu et al., 2010; Warrier et al., 2014). These elevated χ_lf_ values are primarily attributed to vehicular emissions, construction dust, and other anthropogenic activities. The χ_fd_% ranged from 0.23 to 5.69% (mean = 1.03%), indicative of the predominance of larger multi-domain (MD) or coarse single-domain (SD) magnetic grains. A negative correlation between χ_lf_ and χ_fd_% (r = –0.5; p < 0.14; n = 10) suggests pollution-related input (Lu et al., 2007). The mean χ_ARM_ was 0.56 × 10^−5^ m^3^ kg^−1^, reflecting moderate contributions from stable single-domain (SSD) grains.

A strong positive correlation between χ_lf_ and SIRM (r = 0.86; p < 0.016; n = 10) supports a ferrimagnetic mineral source, predominantly magnetite (Fig. 5b). Biplot analysis of $\chi _{{{\text{fd}}}} \% \,vs.\,\chi _{{{\text{ARM}}}} /{\text{SIRM}}$ (Fig. 6a) indicates a mineralogical mixture dominated by MD + PSD grains, with minor influence from SP grains. The S-ratio (~ 0.91) and moderate HIRM (~ 298.4 × 10^−5^ A m^2^ kg^−1^) further confirm a mixed magnetic mineral assemblage sourced from both geogenic and anthropogenic contributions.

When compared with previous studies, Lu et al. (2011) reported an average χ_lf_ of 124.1 × 10^−8^ m^3^ kg^−1^ in urban roadside soils, while Lu et al. (2008) noted peaks up to 597 × 10^−8^ m^3^ kg^−1^ near railways. In contrast, our study’s average χ_lf_ value of 346.1 × 10^−8^ m^3^ kg^−1^ near traffic-heavy zones is considerably higher, reinforcing the influence of vehicular and construction-related pollution. Most earlier investigations have attributed elevated magnetic signals in urban soils to traffic emissions and industrial activity, with typical background levels in parent materials ranging from 10–50 × 10^−8^ m^3^ kg^−1^. The strong χ_lf_–SIRM correlations (r ≈ 0.86–0.94), low χ_fd_%, and the mixed magnetic grain characteristics in this study collectively point to a ferrimagnetic mineral signature of predominantly anthropogenic origin.

Agricultural soils exhibited the lowest χ_lf_ values, averaging 81.4 × 10^−8^ m^3^ kg^−1^, ranging from 6.48 × 10^−8^ to 281 × 10^−8^ m^3^ kg^−1^ (Supplementary Table S5). Paddy soils exhibited lower χ_lf_ values compared to arecanut plantations, reflecting the dissolution of fine magnetic minerals under waterlogged, anoxic conditions (Lu et al., 2012; Maher, 1998). χ_fd_% ranged from 0.96% to 12.34% (mean = 6%), indicating a mixed SP and SSD grain population. χ_ARM_ averaged 0.36 × 10^−5^ m^3^ kg^−1^. Positive correlations between χ_lf_ and SIRM (r = 0.90; p < 0.0003; n = 10) suggest that ferrimagnetic minerals dominate the magnetic properties. S-ratio values (~ 0.88) and HIRM (~ 93.43 × 10^−5^ A m^2^ kg^−1^) point to moderate ferrimagnetic mineral dominance with minimal contributions from hard minerals. The $\chi _{{{\text{fd}}}} \% \,vs.\,\chi _{{{\text{ARM}}}} /{\text{SIRM}}$ biplot indicates a prevalence of coarse SSD and MD grains with minor SP components (Fig. 6a).

Our study, along with findings by Amrutha et al. (2021), reported the lowest magnetic mineral content in agricultural soils, as indicated by low χ_lf_ values (81.4 vs. 92.39 × 10^−8^ m^3^ kg^−1^). Both χ_ARM_ and χ_fd_% values were low to moderate, suggesting a predominance of coarse-grained magnetic particles and limited input from superparamagnetic (SP) grains—likely due to the waterlogged conditions typical of paddy fields. Strong correlations among χ_lf_, SIRM, and HIRM point to the presence of ferrimagnetic minerals, with minor contributions from antiferromagnetic components such as hematite and goethite. Similarly, Wang et al. (2025) reported a broad χ_lf_ range (41.24–462.91 × 10^−8^  m^3^ kg^−1^), with variations closely tied to land use and drainage conditions. In particular, poorly drained sites showed altered wet–dry cycles that significantly influenced magnetic mineral formation.

Industrial soils exhibited moderate χ_lf_ values, averaging 139.34 × 10^−8^ m^3^ kg^−1^, with the highest concentrations near fabrication workshops (IS1 and IS10, Fig. 1). Elevated magnetic susceptibility at industrial sites is attributed to particulate emissions from fabrication and combustion processes (Karimi et al., 2011; Lu et al., 2007). χ_fd_% values suggested coarse SSD grain dominance, while χ_ARM_ values (~ 0.92 × 10^−5^ m^3^ kg^−1^) indicated moderate amounts of fine-grained ferrimagnetic material. Correlations between χ_lf_ and χ_ARM_ (r = 0.98; p < 0.0001; n = 10; Fig. 5a) and IRM_300mT_ (r = 0.98; p < 0.0001; n = 10) highlight the influence of ferrimagnetic minerals. S-ratio values (~ 0.85) and elevated HIRM (~ 283.28 × 10^−5^ A m^2^ kg^−1^) confirm a mixture of soft and hard magnetic minerals. Biplot of $\chi _{{{\text{fd}}}} \% \,vs.\,\chi _{{{\text{ARM}}}} /{\text{SIRM}}$ (Fig. 6a) suggest a moderate anthropogenic impact with SP + SSD contributions.

In our study, industrial soils exhibited elevated χ_lf_ values (average: 139.34 × 10^−8^ m^3^ kg^−1^), which is higher than the values reported by Yang et al. (2012), yet lower than those observed in heavily industrialized regions of Hangzhou as documented by Lu and Bai (2006). Similar to these earlier studies, the low χ_fd_% values in our samples indicate a dominance of coarser magnetic grains, likely originating from industrial emissions. Strong correlations observed between χ_lf_, χ_ARM_, and IRM_300mT_ further corroborate ferrimagnetic mineral input from anthropogenic sources, consistent with the trends noted by Lu and Bai (2006). The S-ratio in our samples (~ 0.85) suggests a mixture of low- and high-coercivity magnetic minerals, aligning with values from industrial soils in Hangzhou (0.73) and slightly lower than those reported by Yang et al., (2012; ~ 0.93). These results collectively support the influence of industrial pollution on the magnetic properties of soils in the study area.

Residential soils exhibited higher χ_lf_ values (average 360.2 × 10^−8^ m^3^ kg^−1^) than roadside, agricultural, and even industrial soils. χ_fd_% averaged 8.32%, indicating a mixture of SP and SSD magnetic grains associated with both natural soil development and anthropogenic inputs (Lu et al., 2007). χ_ARM_ averaged 1.69 × 10^−5^ m^3^ kg^−1^, with strong positive correlations to SIRM (r = 0.87; p < 0.001; n = 10) and IRM_300mT_ (r = 0.89; p < 0.005; n = 10). S-ratio values (~ 0.90) and HIRM (~ 190.49 × 10^−5^ A m^2^ kg^−1^) suggest dominance of soft ferrimagnetic minerals, with localized hematite/goethite enrichment from construction activities. The $\chi _{{{\text{ARM}}}} /\chi _{{{\text{lf}}}} \,vs.\,\chi _{{{\text{ARM}}}} /\chi _{{{\text{fd}}}}$ show that residential soils fall within the “soil, paleosoils, and catchment-derived sediments” field with minor anthropogenic influence, lacking bacterial magnetite contributions (Fig. 6b).

Soils from the residential area exhibit strong pedogenic influence, characterized by relatively high χ_fd_% and moderate χ_ARM_, SIRM, and HIRM values. This contrasts with findings from Maity et al. (2022) in West Bengal, India, and Wang et al. (2014) in northwestern China, where residential soils reflected clearer anthropogenic influence—marked by higher χ_lf_, SIRM, and HIRM values but low χ_fd_% (< 3%), indicative of coarse, pollution-derived magnetite. In contrast, Lu et al. (2007b) reported a mixture of SP and SSD grains with moderate χ_fd_% (~ 8.32%) in urban soils. Compared to these studies, the residential soils in our study area are distinguished by a greater presence of fine, superparamagnetic (SP) grains and minimal influence from industrial or traffic-related pollution sources.

### Assessment of potentially toxic elements (PTEs) concentrations and soil contamination

Potentially toxic elements (PTEs) concentrations and associated contamination indices were analysed across different land-use types to evaluate the influence of natural and anthropogenic factors (Table 2). In forest soils (Supplementary Table S7), iron (Fe) was the most abundant element, with an average concentration of 79,018 mg/kg and a maximum of 118,688 mg/kg. Manganese (Mn) followed, with a mean concentration of 1,017 mg/kg. Chromium (Cr) exhibited high variability, with a mean of 998 mg/kg and a maximum of 4,919 mg/kg. Nickel (Ni), zinc (Zn), and copper (Cu) occurred at moderate levels, while lead (Pb) was the least abundant (mean: 20.87 mg/kg). The order of PTEs abundance was Fe > Mn > Cr > Ni > Zn > Cu > Pb. These values reflect the natural geochemical enrichment of Fe and Mn typical of lateritic soils (Hernandez et al., 2003; Sarkar et al., 2020). However, the wide range in Cr concentrations may indicate localized anthropogenic inputs (Satapathy & Panda, 2018). Although all measured metal concentrations remained within permissible limits (World Health Organization, 1996), the long-term toxicity of Pb remains a concern (Bouida et al., 2022).

**Table 2:** Mean values of Contamination Factor (CF), Geoaccumulation Index (Igeo), Enrichment Factor (EF), and Pollution Load Index (PLI) for major and trace elements across different land-use categories in the Manipal region: Industrial Site (IS), Roadside Site (RoS), Agricultural Site (AS), and Residential Site (ReS)

	Contamination factor (CF)				Geoaccumulation index (Igeo)				Enrichment Factor (EF)				Pollution Load Index (PLI)			
	IS mean	RoS mean	AS mean	ReS mean	IS mean	RoS mean	AS mean	ReS mean	IS mean	RoS mean	AS mean	ReS mean	IS mean	RoS mean	AS mean	ReS mean
Al	0.93	0.75	1.03	1.19	− 0.69	− 1	− 0.55	− 0.34	1	1	1	1	1.36	0.92	0.92	0.89
B	0.75	0.52	1.12	0.77	− 1	− 1.52	− 0.42	− 0.96	0.81	0.7	1.09	0.65				
Ba	2.17	2.4	1.09	1.23	0.54	0.68	− 0.46	− 0.29	2.34	3.2	1.06	1.03				
Ca	4.93	6.04	0.51	1.63	1.72	2.01	− 1.56	0.12	5.3	8.08	0.5	1.37				
Cr	0.56	0.23	0.71	0.45	− 1.41	− 2.7	− 1.08	− 1.74	0.61	0.31	0.69	0.38				
Cu	3.11	0.78	1.21	0.69	1.05	− 0.95	− 0.31	− 1.13	3.34	1.04	1.18	0.58				
Fe	0.71	0.45	0.82	0.97	− 1.08	− 1.74	− 0.87	− 0.62	0.76	0.6	0.8	0.82				
K	1.92	2.66	1.25	1.3	0.36	0.83	− 0.26	− 0.2	2.07	3.55	1.22	0.82				
Mg	1.24	1	0.71	0.88	− 0.27	− 0.58	− 1.09	− 0.77	1.33	1.34	0.69	0.74				
Mn	0.49	0.26	0.43	0.39	− 1.6	− 2.51	− 1.8	− 1.96	0.53	0.35	0.42	0.32				
Ni	0.57	0.31	0.78	0.58	− 1.4	− 2.26	− 0.94	− 1.38	0.61	0.42	0.76	0.49				
Pb	6.26	2.15	1.37	1.46	2.06	0.52	− 0.13	− 0.04	6.74	2.87	1.34	1.23				
Zn	1.35	1.57	1.95	1.14	− 0.16	0.07	0.38	− 0.4	1.45	2.1	1.9	0.96				

CF values > 1 indicate moderate to considerable contamination; positive Igeo values suggest anthropogenic enrichment; EF values > 1.5 signify moderate enrichment from non-crustal sources. PLI values > 1 represent overall pollution stress, particularly evident in the industrial site

Roadside soils (Supplementary Table S7) also showed Fe as the dominant element (average: 35,542 mg/kg). However, Zn and Cr exhibited higher concentrations than Mn, Cu, Pb, and Ni. Notably, elevated concentrations of Zn, Pb, and Ni were recorded at RoS2, a high-traffic junction (Fig. 1), corroborating previous findings that associate such levels with vehicular emissions, tire wear, and road runoff (Akhter & Madany, 1993; Al-Khashman, 2004). Calcium (Ca) concentrations were also highest in roadside soils (12,271 mg/kg), likely originating from concrete and construction materials, as reported by Fergusson and Kim (1991). Enrichment factor (EF) values indicated moderate enrichment for Pb (2.87) and Zn (2.09). Geoaccumulation indices (I_geo_) and contamination factor (CF) values suggested low to moderate pollution levels for Pb and Zn. The Pollution Load Index (PLI) was 0.92, indicative of background contamination levels (Table 2).

Agricultural soils exhibited high Fe concentrations (average: 64,652 mg/kg), followed by Cr, Mn, Zn, Ni, Cu, and Pb (Supplementary Table S7). At AS7 (paddy fields), Cr and Ni levels exceeded permissible limits, likely due to fertilizer application and waterlogging (Reddy et al., 2013; Solgi et al., 2018). Additional sources could include irrigation water contaminated with domestic effluents (Nagajyoti et al., 2010; Rai et al., 2011) or runoff from upstream industrial zones (Deka & Bhattacharyya, 2009). In other agricultural locations, metal levels were within permissible limits. EF values were < 2 for all metals, indicating minimal enrichment. I_geo_ values suggested uncontaminated conditions, though CF values pointed to moderate contamination by Pb and Zn. The PLI remained low at 0.92, implying minor anthropogenic influence (Table 2).

Industrial soils displayed considerable spatial variability. Iron remained the most abundant element (average: 56,209 mg/kg), while Cr, Mn, and Cu were present in substantial concentrations (Supplementary Table S7). Chromium concentrations at IS1 exceeded the Canadian Soil Quality Guidelines (Dhakate et al., 2020), likely due to discharges from fabrication and chemical units (Govil et al., 2008; Ramesh Kumar & Anbazhagan, 2018). Elevated Cu and Pb concentrations at IS3 may be attributed to untreated effluents from a nearby laboratory (Lu et al., 2011) or packaging industries (Gisbert et al., 2003; Seaward & Richardson, 1990). Nickel concentrations were comparable to those reported in the Thane–Belapur industrial corridor, where industrial effluents are a major source (Krishna & Govil, 2005). Zinc concentrations, although moderate, also showed evidence of industrial influence (Dhakate et al., 2020). EF values indicated moderate to significant enrichment for Pb (6.73) and Cu (3.34). I_geo_ values suggested moderate contamination for both. CF values reflected considerable pollution (Pb: 6.2; Cu: 3.1), and the PLI value of 1.35 confirmed significant pollution in industrial soils (Table 2).

Residential soils recorded high Fe concentrations (average: 76,919 mg/kg), likely due to the lateritic parent material (Anand & Gilkes, 1987). Chromium, Mn, Zn, and Ni occurred at moderate levels, whereas Cu and Pb concentrations were relatively low (Supplementary Table S7). Contaminant sources may include atmospheric deposition, vehicle emissions, construction debris, and urban runoff (Alloway, 2013; Chen et al., 2008). Despite diffuse anthropogenic influences, all metal concentrations were within WHO limits (World Health Organization, 1996). CF values indicated moderate contamination by Pb (1.45) and Zn (1.13). The PLI was 0.89, suggesting minimal pollution in residential areas (Table 2).

### Soil magnetic, physico-chemical, and contamination patterns revealed by principal component analysis (PCA)

**Fig. 7:**
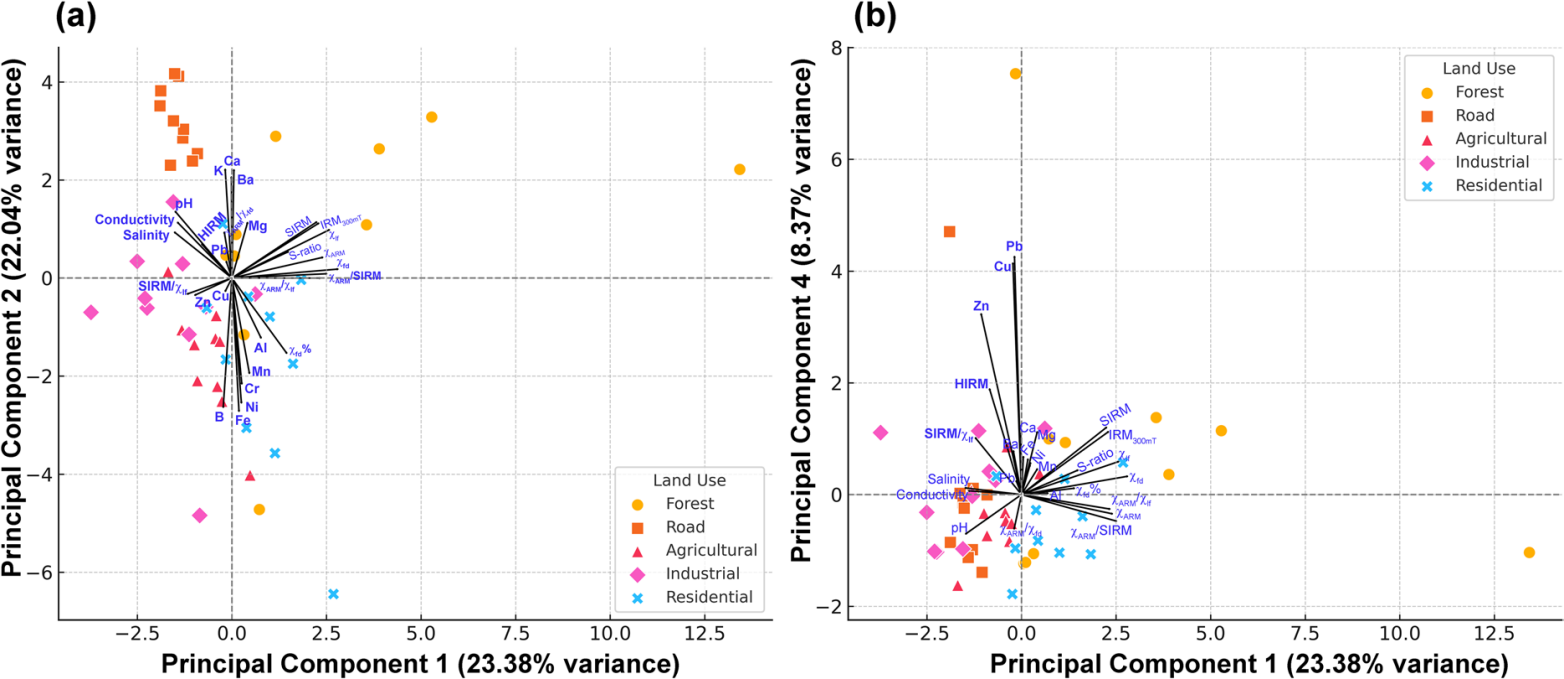
Principal Component Analysis (PCA) biplots illustrating the distribution of soil samples from different land-use types based on physico-chemical parameters, magnetic properties, and elemental concentrations. (**a**) Biplot of Principal Components 1 and 2, explaining 23.38% and 22.14% of the total variance, respectively; (**b**) Biplot of Principal Components 1 and 4, explaining 23.38% and 8.37% of the total variance, respectively. Arrows represent variable loadings, and symbols indicate land-use categories: forest, roadside, agricultural, industrial, and residential soils

Bartlett’s test yielded a highly significant result (χ^2^ = 4507.6, df = 378, p < 0.001), indicating strong correlations among variables and justifying the application of PCA. However, the KMO value of 0.5965 suggests mediocre sampling adequacy, implying that while PCA is appropriate, the results should be interpreted with caution. PCA of the standardized soil dataset revealed distinct patterns of variability across land-use types in the Manipal region (Supplementary Table S8; Fig. 7). The first two principal components, PC1 and PC2, together accounted for a substantial proportion of the total variance, effectively reducing data dimensionality while preserving key environmental signals (Fig. 7a). PC1 explained 23.39% of the variance and was strongly associated with magnetic properties (χ_lf_, χ_ARM_, SIRM, IRM_300mT_), while PC2 accounted for 22.04% of the variance and reflected soil physico-chemical attributes such as pH, electrical conductivity, salinity, and Ca concentrations. PC3 (12.71%) and PC4 (8.37%) further contributed to the model, capturing secondary magnetic variations and patterns of PTEs contamination (Cu, Pb, Zn), respectively. Altogether, the first six components explained approximately 78.7% of the total variance, highlighting the robustness of the PCA model for environmental interpretation.

PC1 serves as a proxy for pedogenic magnetic mineral development, reflecting processes such as organic matter decomposition and microbial activity, which are key to soil formation. In contrast, PC2 captures chemical attributes essential for nutrient availability, cation exchange capacity, and overall ecosystem functioning. The predominantly acidic nature of these soils (except roadside soils) likely facilitates Ca leaching, a common phenomenon in low-pH environments (McLean, 1975).

The PCA biplot (Fig. 7) reveals clear clustering by land-use type. Forest soils group towards the positive end of PC1, indicative of strong pedogenic magnetic enhancement and minimal anthropogenic disturbance. In contrast, roadside and industrial soils plot negatively along both PC1 and PC2, showing strong associations with PTEs (Pb, Zn, Cr, Cu, and Ni) and reflecting contamination from vehicular emissions, construction debris, and industrial discharges. These elements, especially Pb, Zn, and Cr are persistent, non-biodegradable pollutants known to accumulate in urban soils and pose long-term ecological concerns (Gupta, 2020). Agricultural soils exhibit intermediate characteristics, influenced by both natural pedogenic processes and anthropogenic inputs such as fertilizers and irrigation. Residential soils displayed mixed signatures, overlapping with agricultural and industrial clusters, likely due to urban runoff and domestic waste inputs.

A complementary PCA biplot of PC1 and PC4 provided deeper insights into the dual impacts of natural processes and anthropogenic contamination (Fig. 7b). While PC1 continued to represent magnetic mineralogical development, PC4 was driven predominantly by PTEs enrichment, particularly of Cu, Pb, and Zn. Forest soils (e.g., FS5, FS7, FS9) were positioned along high PC1 but low PC4 scores, confirming pristine magnetic properties with minimal contamination. In contrast, roadside and industrial soils (e.g., RoS2, RoS3, IS3) exhibited elevated PC4 scores, suggesting localized PTEs pollution likely linked to anthropogenic activities. Agricultural and residential soils clustered near the origin, indicating moderate degrees of anthropogenic influence.

These findings have important environmental implications. Soils with low magnetic enhancement (low PC1) and high PTE concentrations (high PC4) represent environmentally stressed zones, vulnerable to impaired nutrient cycling, reduced microbial communities, and declining soil health. Thus, PCA not only aids in source differentiation but also serves as a powerful diagnostic tool for environmental monitoring, land-use planning, and soil management. From a practical perspective contamination hotspots identified through PCA may benefit from targeted remediation strategies such as phytoremediation, organic matter amendments, liming, and stricter regulation of urban and industrial runoff. Such interventions can enhance soil resilience and promote ecological stability across the region.

### Environmental implications

This integrated study combining physico-chemical, geochemical, magnetic, and multivariate (PCA) analyses revealed distinct patterns of soil quality degradation associated with different land-use practices in the Manipal region. Forest soils consistently exhibited characteristics of natural pedogenesis, with low contamination levels and strong magnetic mineral development. In contrast, roadside and industrial soils showed clear signatures of anthropogenic influence, including PTE accumulation and altered magnetic properties, consistent with urbanization, vehicular emissions, and industrial activities. Agricultural soils reflected moderate anthropogenic modification, particularly through fertilizer usage and potential irrigation contamination, whereas residential soils displayed mixed signals, indicative of diffuse urban runoff sources. Elevated levels of certain potentially toxic elements (PTEs) can disrupt the geochemical cycles of essential nutrients such as nitrogen, phosphorus, and potassium by interfering with microbial activity and altering nutrient transformation processes. In affected areas, this disruption leads to reduced long-term soil fertility and diminished nutrient use efficiency (Tian et al., 2025). Environmental magnetism emerged as a reliable proxy for distinguishing natural from polluted soils, while PTE indices confirmed localized pollution hotspots. The strong agreement between physico-chemical deterioration, magnetic enhancement, and metal enrichment highlights the need for targeted soil conservation and pollution mitigation strategies, particularly for urban and peri-urban landscapes.

## Conclusions

This study presents a comprehensive assessment of soil quality across diverse land-use types—forest, roadside, agricultural, industrial, and residential—in the Manipal region using a multi-proxy approach combining physico-chemical, geochemical, magnetic, and multivariate statistical methods. The results reveal significant spatial variability in soil properties influenced by land-use practices and anthropogenic inputs.

Forest soils maintained low salinity, acidic pH, and higher frequency-dependent magnetic susceptibility (χ_fd_%), reflecting stable pedogenic processes and minimal human interference. In contrast, roadside and industrial soils displayed elevated electrical conductivity, alkaline pH, and accumulation of potentially toxic elements (PTEs) such as Pb, Zn, Cr, and Cu, indicative of contamination from vehicular and industrial activities. Agricultural soils, particularly in paddy fields, showed signs of acidification and reduced magnetic signals, likely due to fertilizer application and anoxic conditions. Residential soils were moderately impacted, with variable PTE levels and mixed magnetic mineral assemblages.

Magnetic proxies, especially χ_lf_ and χ_fd_%, proved effective in distinguishing between natural and contaminated soils. Their strong correlation with PTE concentrations underscores the utility of environmental magnetism as a cost-effective, non-invasive tool for soil pollution monitoring. Principal Component Analysis (PCA) and contamination indices (CF, Igeo, EF, and PLI) further delineated the clustering of land-use types, with industrial soils showing the highest pollution load index.

By integrating geochemical and magnetic datasets, this study demonstrates that anthropogenic activities are key drivers of soil degradation in urban and peri-urban landscapes. The findings provide essential baseline data to inform land-use planning, environmental regulation, and remediation strategies. While this study offers valuable insights, future research should incorporate seasonal variability, deeper soil horizons, and biological indicators (e.g., microbial and plant health) to develop a holistic understanding of soil ecosystem dynamics and resilience.

## Supplementary Information

Below is the link to the electronic supplementary material.

Supplementary file1 (DOCX 134 kb)

## Data Availability

The raw data used in this study is included in the Supplementary file.
