# Supplementary material for: Tracking soil health and potentially toxic elements (PTEs) across land-use types using physico-chemical, magnetic, and geochemical proxies: a case study from Manipal, Southwestern India
Source: Environ Geochem Health. 2025 Aug 19;47(9):397. doi: 10.1007/s10653-025-02665-9 (PMC12364988; doi:10.1007/s10653-025-02665-9)
Supplement: Supplementary file 1 — Supplementary file1 (DOCX 134 kb) [file 10653_2025_2665_MOESM1_ESM.docx]

**Tracking Soil Health and Potentially Toxic Element (PTE) Pollution Across Land-Use Types Using Physico-Chemical, Magnetic, and Geochemical Proxies: A Case Study from Manipal, Southwestern India**

Jai Vishnu Degvekar^1,#^, Esha Ulhas Gadekar^1,#^, Darshana O.^1^, Jagath Chand^2^, Vadakkeveedu Narayan Amrish^2^, Santhosh Prabhu^2^, Jithin Jose^3^, K. Priya^3^, Anish Kumar Warrier^4,*^

^1^Department of Sciences, Manipal Institute of Technology, Manipal Academy of Higher Education, Manipal, 576104, Karnataka, India

^2^Department of Civil Engineering, Manipal Institute of Technology, Manipal Academy of Higher Education, Manipal, 576104, Karnataka, India

^3^Department of Marine Geology, Mangalore University, Mangalagangothri – 574199, Karnataka, India.

^4^Centre for Climate Studies, Department of Civil Engineering, Manipal Institute of Technology, Manipal Academy of Higher Education, Manipal, 576104, Karnataka, India.

^#^Jai and Esha contributed equally to this work.

^*^Corresponding author: Anish Kumar Warrier – [anish.warrier@manipal.edu](mailto:anish.warrier@manipal.edu)

**The file contains tables containing raw dataset used in this study.**

**Supplementary Table S1.** Sample ID along with the geographic coordinates of each location.

| **Sample ID** | **Latitutde** | **Longitude** | **Sample ID** | **Latitutde** | **Longitude** | **Sample ID** | **Latitutde** | **Longitude** | **Sample ID** | **Latitutde** | **Longitude** | **Sample ID** | **Latitutde** | **Longitude** |
| --- | --- | --- | --- | --- | --- | --- | --- | --- | --- | --- | --- | --- | --- | --- |
| **RoS1** | 13°20'52.49" | 74°46'36.76" | **AS1** | 13°20'11.94" | 74°47'42.42" | **FS1** | 13°20'13.72" | 74°47'37.61" | **IS1** | 13°20'10.83" | 74°47'20.07" | **ReS1** | 13°21'32.13" | 74°47'33.76" |
| **RoS2** | 13°20'50.86" | 74°47'02.17" | **AS2** | 13°19'34.37" | 74°46'50.85" | **FS2** | 13°20'3.60" | 74°47'50.08" | **IS2** | 13°20'09.44" | 74°47'17.59" | **ReS2** | 13°20'22.82" | 74°47'28.03" |
| **RoS3** | 13°20'56.59" | 74°47'07.79" | **AS3** | 13°21'49.66" | 74°47'55.39" | **FS3** | 13°19'39.68" | 74°47'49.98" | **IS3** | 13°20'13.31" | 74°47'10.02" | **ReS3** | 13°20'26.39" | 74°46'53.70" |
| **RoS4** | 13°20'52.83" | 74°47'15.30" | **AS4** | 13°21'50.55" | 74°47'53.02" | **FS4** | 13°21'36.01" | 74°47'44.99" | **IS4** | 13°20'03.80" | 74°47'15.35" | **ReS4** | 13°20'44.35" | 74°47'05.36" |
| **RoS5** | 13°21'08.96" | 74°47'12.45" | **AS5** | 13°21'10.65" | 74°48'09.28" | **FS5** | 13°22'3.46" | 74°47'48.32" | **IS5** | 13°20'01.62" | 74°47'06.32" | **ReS5** | 13°20'57.04" | 74°46'34.32" |
| **RoS6** | 13°21'31.0" | 74°47'06.9" | **AS6** | 13°21'12.19" | 74°48'07.71" | **FS6** | 13°20'47.84" | 74°48'1.42" | **IS6** | 13°19'56.1" | 74°47'01.2" | **ReS6** | 13°21'37" | 74°47'14.21" |
| **RoS7** | 13°20'47.25" | 74°46'49.45" | **AS7** | 13°22'05.43" | 74°47'40.85" | **FS7** | 13°20'22.99" | 74°46'40.31" | **IS7** | 13°19'57.41" | 74°47'14.35" | **ReS7** | 13°21'12.47" | 74°46'59.38" |
| **RoS8** | 13°20'37.05" | 74°47'25.82" | **AS8** | 13°20'19.04" | 74°46'32.33" | **FS8** | 13°21'9.24" | 74°46'33.84" | **IS8** | 13°19'52.94" | 74°47'19.57" | **ReS8** | 13°21'04.28" | 74°47'51.29" |
| **RoS9** | 13°21'12.68" | 74°47'28.10" | **AS9** | 13°21'57.55" | 74°47'34.57" | **FS9** | 13°22'19.77" | 74°46'57.78" | **IS9** | 13°20'03.57" | 74°47'22.06" | **ReS9** | 13°21'32.76" | 74°47'58.61" |
| **RoS10** | 13°21'19.44" | 74°46'54.52" | **AS10** | 13°21'32.75" | 74°46'22.48" | **FS10** | 13°21'51.90" | 74°47'28.46" | **IS10** | 13°20'06.26" | 74°47'05.14" | **ReS10** | 13°19'48.55" | 74°47'38.31" |

**Supplementary Table S2**. Pollution assessment indices used in this study, including their definitions, classification thresholds, and reference values. The indices comprise the Contamination Factor (CF), Pollution Load Index (PLI) (Tomlinson et al., 1980), Geoaccumulation Index (Igeo) (Pathak et al., 2015a), and Enrichment Factor (EF) (Loska et al., 2004), which collectively aid in evaluating the extent and sources of soil contamination.

| **Environmental Pollution Indices** | **Level of contamination** | **Numerical Value** |
| --- | --- | --- |
| **Contamination Factor (CF)** | Low | CF < 1 |
|  | Moderate | 1 ≤ CF ≤ 3 |
|  | Considerable | 3 ≤ CF ≤ 6 |
|  | Very high | CF > 6 |
| **Pollution Load Index (PLI)** | Low pollution | PLI <1 |
|  | Increased pollution | PLI >1 |
| **Geoaccumulation Index (I_geo_)** | Unpolluted | I_geo_ ≤ 0 |
|  | Unpolluted to moderately polluted | 0 < I_geo_ ≤ 1 |
|  | Moderately polluted | 1 < I_geo_ ≤ 2 |
|  | Moderately to heavily polluted | 2 < I_geo_ ≤ 3 |
|  | Heavily polluted | 3 < I_geo_ ≤ 4 |
|  | Heavily to extremely polluted | 4 < I_geo_ ≤ 5 |
|  | Extremely polluted | I_geo_ ≥ 5 |
| **Enrichment Factor (EF)** | Deficiency to minimal enrichment | EF < 2 |
|  | Moderate enrichment | 2 ≤ EF ≤ 5 |
|  | Significant enrichment | 5 ≤ EF ≤ 20 |
|  | Very high enrichment | 20 ≤ EF ≤ 40 |
|  | Extremely high enrichment | EF > 40 |

**Supplementary Table S3.** Summary table of Munsell soil colour for different land use types.

| **Forest Soil** | | **Road Soil** | | **Agricultural soil** | | **Industrial soil** | | **Residential soil** | |
| --- | --- | --- | --- | --- | --- | --- | --- | --- | --- |
| **Sample ID** | **Munsell color index** | **Sample ID** | **Munsell color index** | **Sample ID** | **Munsell color index** | **Sample ID** | **Munsell color index** | **Sample ID** | **Munsell color index** |
| **FS1** | 2.5Y- 2.5/1 black | **RoS1** | 2.5Y- 7/1 light gray | **AS1** | 2.5Y- 5/2. greyish brown | **IS1** | 10R- 3/2 dusky red | **ReS1** | 2.5Y- 2.5/1 black |
| **FS2** | 2.5YR- 3/1 very dark reddish gray | **RoS2** | 2.5Y- 6/1 gray | **AS2** | 2.5Y- 5/1 grey | **IS2** | 5YR- 2.5/2 dark redish brown | **ReS2** | 2.5Y- 3/3 dark olive brown |
| **FS3** | 2.5YR- 3/2 dusky red | **RoS3** | 2.5Y- 5/2 grayish brown | **AS3** | 2.5Y- 3/2 very dark greyish brown | **IS3** | 2.5Y- 3/1 very dark grey | **ReS3** | 7.5YR- 3/3 dark brown |
| **FS4** | 2.5Y- 3/2 very dark greyish brown | **RoS4** | 2.5Y- 4/2 dark grayish brown | **AS4** | 5 YR- 2.5/2 dark reddish brown | **IS4** | 2.5Y- 2.5/1 black | **ReS4** | 7.5YR- 3/2 dark brown |
| **FS5** | 7.5YR- 2.5/2 very dark brown | **RoS5** | 2.5Y- 5/1 gray | **AS5** | 7.5YR- 3/2 dark brown | **IS5** | 7.5YR- 2.5/2 very dark brown | **ReS5** | 7.5YR- 2.5/3 very dark brown |
| **FS6** | 2.5Y- 4/3 olive brown | **RoS6** | 2.5Y- 6/1 gray | **AS6** | 2.5Y- 4/2 dark greyish brown | **IS6** | 7.5YR- 3/2 dark brown | **ReS6** | 2.5Y- 3/3 dark olive brown |
| **FS7** | 2.5Y- 4/2 dark greyish brown | **RoS7** | 2.5Y- 6/1 gray | **AS7** | 2.5Y- 4/3 olive brown | **IS7** | 10R- 4/8 red | **ReS7** | 10YR- 4/4 dark yellowish brown |
| **FS8** | 2.5YR- reddish brown | **RoS8** | 2.5Y- 7/1 light gray | **AS8** | 2.5Y- 4/2 dark greyish brown | **IS8** | 2.5Y- 5/2 greyish brown | **ReS8** | 7.5YR- 3/3 dark brown |
| **FS9** | 2.5YR- 4/3 reddish brown | **RoS9** | 2.5Y- 5/2 grayish brown | **AS9** | 2.5Y- 5/2 greyish brown | **IS9** | 2.5Y- 4/2 dark greyish brown | **ReS9** | 10YR- 3/2 very dark greyish brown |
| **FS10** | 7.5YR- 4/3 brown | **RoS10** | 2.5Y- 5/2 grayish brown | **AS10** | 2.5Y- 4/2 dark greyish brown | **IS10** | 2.5Y- 3/3 dark olive brown | **ReS10** | 7.5YR- 3/4 dark brown |

**Supplementary Table S4.** Physico-chemical parameters of each soil type showing average value, maximum value, minimum value, standard deviation and CV% (Coefficient Variable).

| **Sample ID** | **pH (1:3)** | **Conductivity (μS/cm)** | **Salinity (‰)** |
| --- | --- | --- | --- |
| **FS1** | 6.20 | 17.45 | 0.010 |
| **FS2** | 5.62 | 8.92 | 0.000 |
| **FS3** | 5.05 | 21.39 | 0.010 |
| **FS4** | 5.77 | 27.90 | 0.010 |
| **FS5** | 5.21 | 15.10 | 0.000 |
| **FS6** | 5.48 | 11.51 | 0.000 |
| **FS7** | 5.72 | 10.80 | 0.000 |
| **FS8** | 5.45 | 4.73 | 0.000 |
| **FS9** | 5.46 | 18.71 | 0.000 |
| **FS10** | 5.06 | 11.37 | 0.000 |
| **FS mean** | 5.50 | 14.79 | 0.003 |
| **Max** | 6.20 | 27.90 | 0.010 |
| **Min** | 5.05 | 4.73 | 0.000 |
| **Std dev** | 0.35 | 6.77 | 0.005 |
| **CV%** | 6.38 | 45.75 | 161.015 |
|  | | | |
| **Sample ID** | **pH (1:3)** | **Conductivity (μS/cm)** | **Salinity (‰)** |
| **RoS 1** | 8.92 | 55.7 | 0.020 |
| **RoS 2** | 8.74 | 99.7 | 0.040 |
| **RoS 3** | 8.59 | 45.1 | 0.020 |
| **RoS 4** | 8.35 | 66.3 | 0.030 |
| **RoS 5** | 8.44 | 51.8 | 0.020 |
| **RoS 6** | 8.75 | 53.5 | 0.020 |
| **RoS 7** | 8.01 | 27.1 | 0.010 |
| **RoS 8** | 9.07 | 97.3 | 0.040 |
| **RoS 9** | 8.67 | 65.5 | 0.030 |
| **RoS 10** | 9.28 | 71.3 | 0.030 |
| **RoS mean** | 8.68 | 63.33 | 0.026 |
| **Max** | 9.28 | 99.70 | 0.040 |
| **Min** | 8.01 | 27.10 | 0.010 |
| **Std dev** | 0.37 | 22.33 | 0.010 |
| **CV%** | 4.21 | 35.26 | 37.157 |
|  | | | |
| **Sample ID** | **pH (1:3)** | **Conductivity (μS/cm)** | **Salinity (‰)** |
| **AS1** | 5.68 | 15.91 | 0.010 |
| **AS2** | 6.64 | 20.89 | 0.010 |
| **AS3** | 6.09 | 28 | 0.010 |
| **AS4** | 6.44 | 16.06 | 0.010 |
| **AS5** | 5.77 | 6.5 | 0.000 |
| **AS6** | 6.34 | 21.59 | 0.010 |
| **AS7** | 6.1 | 20.07 | 0.010 |
| **AS8** | 5.58 | 9.81 | 0.000 |
| **AS9** | 5.88 | 14.34 | 0.000 |
| **AS10** | 6.07 | 33.7 | 0.010 |
| **AS mean** | 6.06 | 18.69 | 0.007 |
| **Max** | 6.64 | 33.70 | 0.010 |
| **Min** | 5.58 | 6.50 | 0.000 |
| **Std dev** | 0.34 | 8.08 | 0.005 |
| **CV%** | 5.65 | 43.26 | 69.007 |
|  | | | |
| **Sample ID** | **pH (1:3)** | **Conductivity (μS/cm)** | **Salinity (‰)** |
| **IS 1** | 5.59 | 30.08 | 0.010 |
| **IS 2** | 7.27 | 121.7 | 0.050 |
| **IS 3** | 7.68 | 30.3 | 0.010 |
| **IS 4** | 7.92 | 110.1 | 0.050 |
| **IS 5** | 7.84 | 38.7 | 0.020 |
| **IS 6** | 7.83 | 36.6 | 0.010 |
| **IS 7** | 5.57 | 20.49 | 0.010 |
| **IS 8** | 8.3 | 93.6 | 0.040 |
| **IS 9** | 8.59 | 105.2 | 0.050 |
| **IS 10** | 7.84 | 144.7 | 0.060 |
| **IS mean** | 7.44 | 73.15 | 0.031 |
| **Max** | 8.59 | 144.70 | 0.060 |
| **Min** | 5.57 | 20.49 | 0.010 |
| **Std dev** | 1.04 | 46.28 | 0.021 |
| **CV%** | 14.00 | 63.27 | 67.064 |
|  | | | |
| **Sample ID** | **pH (1:3)** | **Conductivity (μS/cm)** | **Salinity (‰)** |
| **ReS 1** | 7.42 | 35.6 | 0.010 |
| **ReS 2** | 6.51 | 9.04 | 0.000 |
| **ReS 3** | 7.7 | 64.8 | 0.030 |
| **ReS 4** | 6.74 | 16.15 | 0.010 |
| **ReS 5** | 6.18 | 6.84 | 0.000 |
| **ReS 6** | 6.42 | 42.3 | 0.020 |
| **ReS 7** | 6.69 | 14.7 | 0.000 |
| **ReS 8** | 5.9 | 12.59 | 0.000 |
| **ReS 9** | 6.49 | 26.4 | 0.010 |
| **ReS 10** | 6.22 | 10.35 | 0.000 |
| **ReS mean** | 6.63 | 23.88 | 0.008 |
| **Max** | 7.70 | 64.80 | 0.030 |
| **Min** | 5.90 | 6.84 | 0.000 |
| **Std dev** | 0.55 | 18.63 | 0.010 |
| **CV%** | 8.37 | 78.01 | 129.099 |

**Supplementary Table S5**. Magnetic parameters of different soil types.

| **Sample ID** | **XLF (10^-8^m^3^kg^-1^)** | **XARM (10^-5^m^3^kg^-1^)** | **IRM300** | **SIRM(10^-5^Am^2^kg^-1^)** | **S-ratio** | **HIRM (10^-5^ Am^2^ kg^-1^)** | **SIRM/Xlf** | **Xarm/Xlf** | **XARM/SIRM (10⁻³ m/A)** | **XFD (10^-8^m^3^kg^-1^)** | **XFD%** | **XARM/XFD** |
| --- | --- | --- | --- | --- | --- | --- | --- | --- | --- | --- | --- | --- |
| **FS1** | 278.00 | 1.50 | 2755.61 | 2848.86 | 0.97 | 93.24 | 10.25 | 5.39 | 52.56 | 21.00 | 7.55 | 71.31 |
| **FS2** | 158.00 | 1.12 | 1423.64 | 1624.07 | 0.88 | 200.43 | 10.28 | 7.08 | 68.88 | 13.00 | 8.23 | 86.06 |
| **FS3** | 325.00 | 2.08 | 2872.64 | 3260.21 | 0.88 | 387.57 | 10.03 | 6.40 | 63.83 | 24.00 | 7.38 | 86.71 |
| **FS4** | 519.00 | 2.67 | 5838.57 | 6143.49 | 0.95 | 304.92 | 11.84 | 5.14 | 43.45 | 29.00 | 5.59 | 92.04 |
| **FS5** | 1650.00 | 8.14 | 16931.44 | 17067.01 | 0.99 | 135.57 | 10.34 | 4.93 | 47.70 | 80.00 | 4.85 | 101.76 |
| **FS6** | 25.80 | 0.19 | 193.58 | 222.00 | 0.87 | 28.43 | 8.60 | 7.45 | 86.60 | 2.20 | 8.53 | 87.39 |
| **FS7** | 2140.00 | 115.87 | 16109.60 | 16282.47 | 0.99 | 172.87 | 7.61 | 54.15 | 711.64 | 180.00 | 8.41 | 643.74 |
| **FS8** | 19.10 | 0.12 | 116.75 | 138.94 | 0.84 | 22.19 | 7.27 | 6.17 | 84.75 | 1.80 | 9.42 | 65.42 |
| **FS9** | 1060.00 | 6.09 | 7731.26 | 7775.66 | 0.99 | 44.40 | 7.34 | 5.75 | 78.34 | 99.00 | 9.34 | 61.53 |
| **FS10** | 676.00 | 4.89 | 4732.29 | 4737.72 | 1.00 | 5.43 | 7.01 | 7.24 | 103.32 | 93.00 | 13.76 | 52.63 |
| **FS mean** | 685.09 | 14.27 | 5870.54 | 6010.04 | 0.94 | 139.51 | 9.06 | 10.97 | 134.11 | 54.30 | 8.31 | 134.86 |
| **Max** | 2140.00 | 115.87 | 16931.44 | 17067.01 | 1.00 | 387.57 | 11.84 | 54.15 | 711.64 | 180.00 | 13.76 | 643.74 |
| **Min** | 19.10 | 0.12 | 116.75 | 138.94 | 0.84 | 5.43 | 7.01 | 4.93 | 43.45 | 1.80 | 4.85 | 52.63 |
| **Std dev** | 720.14 | 35.80 | 6107.74 | 6121.87 | 0.06 | 128.67 | 1.70 | 15.20 | 203.80 | 57.54 | 2.42 | 179.46 |
| **CV%** | 105.12 | 250.90 | 104.04 | 101.86 | 6.60 | 92.23 | 18.72 | 138.53 | 151.97 | 105.97 | 29.18 | 133.07 |
|  | | | | | | | | | | | | |
| **Sample ID** | **XLF (10^-8^m^3^kg^-1^)** | **XARM (10^-5^m^3^kg^-1^)** | **IRM300** | **SIRM(10^-5^Am^2^kg^-1^)** | **S-ratio** | **HIRM (10^-5^ Am^2^ kg^-1^)** | **SIRM/Xlf** | **Xarm/Xlf** | **XARM/SIRM (10⁻³ m/A)** | **XFD (10^-8^m^3^kg^-1^)** | **XFD%** | **XARM/XFD** |
| **RoS 1** | 426.00 | 0.55 | 3660.35 | 3999.62 | 0.92 | 339.27 | 9.39 | 1.30 | 13.85 | 1.00 | 0.23 | 553.83 |
| **RoS 2** | 431.00 | 0.85 | 5483.26 | 5844.41 | 0.94 | 361.15 | 13.56 | 1.98 | 14.57 | 4.00 | 0.93 | 212.93 |
| **RoS 3** | 321.00 | 0.72 | 3194.74 | 3705.37 | 0.86 | 510.63 | 11.54 | 2.26 | 19.55 | 1.00 | 0.31 | 724.43 |
| **RoS 4** | 582.00 | 0.58 | 4647.49 | 5035.80 | 0.92 | 388.31 | 8.65 | 0.99 | 11.50 | 3.00 | 0.52 | 192.99 |
| **RoS 5** | 380.00 | 0.51 | 2902.13 | 3219.11 | 0.90 | 316.99 | 8.47 | 1.35 | 15.97 | 2.00 | 0.53 | 257.01 |
| **RoS 6** | 162.00 | 0.50 | 1243.25 | 1363.19 | 0.91 | 119.94 | 8.41 | 3.07 | 36.47 | 1.00 | 0.62 | 497.10 |
| **RoS 7** | 158.00 | 0.29 | 1163.51 | 1341.04 | 0.87 | 177.52 | 8.49 | 1.82 | 21.42 | 9.00 | 5.70 | 31.91 |
| **RoS 8** | 350.00 | 0.56 | 2367.49 | 2601.08 | 0.91 | 233.59 | 7.43 | 1.60 | 21.50 | 2.00 | 0.57 | 279.58 |
| **RoS 9** | 332.00 | 0.44 | 2651.99 | 2942.33 | 0.90 | 290.34 | 8.86 | 1.32 | 14.84 | 2.00 | 0.60 | 218.35 |
| **RoS 10** | 319.00 | 0.63 | 2898.73 | 3145.42 | 0.92 | 246.69 | 9.86 | 1.97 | 19.94 | 1.00 | 0.31 | 627.21 |
| **RoS mean** | 346.10 | 0.56 | 3021.29 | 3319.74 | 0.91 | 298.44 | 9.47 | 1.76 | 18.96 | 2.60 | 1.03 | 359.53 |
| **Max** | 582.00 | 0.85 | 5483.26 | 5844.41 | 0.94 | 510.63 | 13.56 | 3.07 | 36.47 | 9.00 | 5.70 | 724.43 |
| **Min** | 158.00 | 0.29 | 1163.51 | 1341.04 | 0.86 | 119.94 | 7.43 | 0.99 | 11.50 | 1.00 | 0.23 | 31.91 |
| **Std dev** | 125.27 | 0.15 | 1347.84 | 1428.77 | 0.02 | 111.79 | 1.81 | 0.60 | 7.05 | 2.46 | 1.65 | 224.81 |
| **CV%** | 36.20 | 27.30 | 44.61 | 43.04 | 2.64 | 37.46 | 19.11 | 33.99 | 37.18 | 94.56 | 160.01 | 62.53 |
|  | | | | | | | | | | | | |
| **Sample ID** | **XLF (10^-8^m^3^kg^-1^)** | **XARM (10^-5^m^3^kg^-1^)** | **IRM300** | **SIRM(10^-5^Am^2^kg^-1^)** | **S-ratio** | **HIRM (10^-5^ Am^2^ kg^-1^)** | **SIRM/Xlf** | **Xarm/Xlf** | **XARM/SIRM (10⁻³ m/A)** | **XFD (10^-8^m^3^kg^-1^)** | **XFD%** | **XARM/XFD** |
| **AS1** | 25.70 | 0.14 | 192.16 | 211.70 | 0.91 | 19.53 | 8.24 | 5.48 | 66.58 | 1.90 | 7.39 | 74.18 |
| **AS2** | 104.00 | 0.41 | 1435.46 | 1519.94 | 0.94 | 84.48 | 14.61 | 3.95 | 27.04 | 1.00 | 0.96 | 411.02 |
| **AS3** | 6.48 | 0.00 | 66.52 | 69.28 | 0.96 | 2.75 | 10.69 | 0.75 | 7.06 | 0.80 | 12.35 | 6.11 |
| **AS4** | 63.40 | 0.36 | 457.90 | 540.67 | 0.85 | 82.78 | 8.53 | 5.62 | 65.93 | 5.90 | 9.31 | 60.41 |
| **AS5** | 101.00 | 0.62 | 1053.30 | 1260.93 | 0.84 | 207.63 | 12.48 | 6.16 | 49.36 | 7.40 | 7.33 | 84.11 |
| **AS6** | 80.70 | 0.30 | 1051.82 | 1247.76 | 0.84 | 195.94 | 15.46 | 3.70 | 23.92 | 3.30 | 4.09 | 90.46 |
| **AS7** | 281.00 | 1.28 | 1931.93 | 2035.39 | 0.95 | 103.46 | 7.24 | 4.55 | 62.87 | 16.00 | 5.69 | 79.97 |
| **AS8** | 32.10 | 0.12 | 461.21 | 539.54 | 0.85 | 78.33 | 16.81 | 3.83 | 22.81 | 2.10 | 6.54 | 58.60 |
| **AS9** | 110.00 | 0.39 | 1082.06 | 1198.20 | 0.90 | 116.14 | 10.89 | 3.58 | 32.88 | 5.00 | 4.55 | 78.79 |
| **AS10** | 9.99 | 0.02 | 88.57 | 111.87 | 0.79 | 23.30 | 11.20 | 1.58 | 14.12 | 0.25 | 2.50 | 63.20 |
| **AS mean** | 81.44 | 0.36 | 782.09 | 873.53 | 0.88 | 91.43 | 11.62 | 3.92 | 37.26 | 4.37 | 6.07 | 100.69 |
| **Max** | 281.00 | 1.28 | 1931.93 | 2035.39 | 0.96 | 207.63 | 16.81 | 6.16 | 66.58 | 16.00 | 12.35 | 411.02 |
| **Min** | 6.48 | 0.00 | 66.52 | 69.28 | 0.79 | 2.75 | 7.24 | 0.75 | 7.06 | 0.25 | 0.96 | 6.11 |
| **Std dev** | 80.36 | 0.38 | 626.67 | 670.87 | 0.06 | 69.24 | 3.22 | 1.71 | 22.21 | 4.72 | 3.31 | 111.57 |
| **CV%** | 98.68 | 103.03 | 80.13 | 76.80 | 6.47 | 75.73 | 27.69 | 43.69 | 59.62 | 108.17 | 54.53 | 110.81 |
|  | | | | | | | | | | | | |
| **Sample ID** | **XLF (10^-8^m^3^kg^-1^)** | **XARM (10^-5^m^3^kg^-1^)** | **IRM300** | **SIRM(10^-5^Am^2^kg^-1^)** | **S-ratio** | **HIRM (10^-5^ Am^2^ kg^-1^)** | **SIRM/Xlf** | **Xarm/Xlf** | **XARM/SIRM (10⁻³ m/A)** | **XFD (10^-8^m^3^kg^-1^)** | **XFD%** | **XARM/XFD** |
| **IS 1** | 375.00 | 2.81 | 5525.57 | 5934.49 | 0.93 | 408.93 | 15.83 | 7.50 | 47.37 | 27.00 | 7.20 | 104.13 |
| **IS 2** | 86.80 | 0.42 | 1558.26 | 2332.80 | 0.67 | 774.54 | 26.88 | 4.78 | 17.80 | 2.70 | 3.11 | 153.79 |
| **IS 3** | 86.80 | 0.51 | 1576.49 | 1777.96 | 0.89 | 201.47 | 20.48 | 5.83 | 28.47 | 1.70 | 1.96 | 297.75 |
| **IS 4** | 101.00 | 0.50 | 1385.31 | 1673.46 | 0.83 | 288.16 | 16.57 | 4.95 | 29.87 | 1.40 | 1.39 | 357.05 |
| **IS 5** | 171.00 | 0.95 | 2429.28 | 2722.29 | 0.89 | 293.01 | 15.92 | 5.58 | 35.03 | 6.00 | 3.51 | 158.95 |
| **IS 6** | 179.00 | 1.30 | 2585.25 | 2830.98 | 0.91 | 245.72 | 15.82 | 7.25 | 45.86 | 11.00 | 6.15 | 118.02 |
| **IS 7** | 39.10 | 0.29 | 435.25 | 610.82 | 0.71 | 175.57 | 15.62 | 7.36 | 47.13 | 3.90 | 9.97 | 73.81 |
| **IS 8** | 57.40 | 0.36 | 637.92 | 758.29 | 0.84 | 120.37 | 13.21 | 6.32 | 47.82 | 1.30 | 2.26 | 278.91 |
| **IS 9** | 98.30 | 0.45 | 1247.91 | 1377.48 | 0.91 | 129.57 | 14.01 | 4.57 | 32.62 | 0.30 | 0.31 | 1497.57 |
| **IS 10** | 199.00 | 1.61 | 2492.77 | 2688.27 | 0.93 | 195.50 | 13.51 | 8.10 | 59.96 | 15.00 | 7.54 | 107.46 |
| **IS mean** | 139.34 | 0.92 | 1987.40 | 2270.68 | 0.85 | 283.28 | 16.78 | 6.22 | 39.19 | 7.03 | 4.34 | 314.74 |
| **Max** | 375.00 | 2.81 | 5525.57 | 5934.49 | 0.93 | 774.54 | 26.88 | 8.10 | 59.96 | 27.00 | 9.97 | 1497.57 |
| **Min** | 39.10 | 0.29 | 435.25 | 610.82 | 0.67 | 120.37 | 13.21 | 4.57 | 17.80 | 0.30 | 0.31 | 73.81 |
| **Std dev** | 98.38 | 0.80 | 1446.04 | 1511.41 | 0.09 | 192.88 | 4.09 | 1.27 | 12.47 | 8.47 | 3.17 | 426.45 |
| **CV%** | 70.60 | 86.80 | 72.76 | 66.56 | 10.76 | 68.09 | 24.38 | 20.40 | 31.82 | 120.51 | 73.12 | 135.49 |
|  | | | | | | | | | | | | |
| **Sample ID** | **XLF (10^-8^m^3^kg^-1^)** | **XARM (10^-5^m^3^kg^-1^)** | **IRM300** | **SIRM(10^-5^Am^2^kg^-1^)** | **S-ratio** | **HIRM (10^-5^ Am^2^ kg^-1^)** | **SIRM/Xlf** | **Xarm/Xlf** | **XARM/SIRM (10⁻³ m/A)** | **XFD (10^-8^m^3^kg^-1^)** | **XFD%** | **XARM/XFD** |
| **ReS 1** | 200.00 | 0.77 | 2288.90 | 2710.58 | 0.84 | 421.68 | 13.55 | 3.84 | 28.37 | 11.00 | 5.50 | 69.91 |
| **ReS 2** | 299.00 | 1.40 | 2810.11 | 3089.76 | 0.91 | 279.64 | 10.33 | 4.67 | 45.23 | 19.00 | 6.35 | 73.55 |
| **ReS 3** | 245.00 | 1.59 | 1462.74 | 1754.20 | 0.83 | 291.46 | 7.16 | 6.51 | 90.86 | 29.00 | 11.84 | 54.96 |
| **ReS 4** | 535.00 | 3.18 | 3686.32 | 3845.36 | 0.96 | 159.04 | 7.19 | 5.95 | 82.71 | 51.00 | 9.53 | 62.37 |
| **ReS 5** | 521.00 | 3.39 | 3415.72 | 3499.60 | 0.98 | 83.88 | 6.72 | 6.52 | 97.00 | 59.00 | 11.32 | 57.54 |
| **ReS 6** | 607.00 | 3.58 | 3975.34 | 4201.81 | 0.95 | 226.47 | 6.92 | 5.90 | 85.22 | 65.00 | 10.71 | 55.09 |
| **ReS 7** | 295.00 | 1.70 | 2130.50 | 2343.07 | 0.91 | 212.57 | 7.94 | 5.76 | 72.51 | 28.00 | 9.49 | 60.68 |
| **ReS 8** | 42.00 | 0.18 | 245.88 | 265.51 | 0.93 | 19.63 | 6.32 | 4.31 | 68.16 | 1.70 | 4.05 | 106.45 |
| **ReS 9** | 174.00 | 0.72 | 1807.76 | 1872.89 | 0.97 | 65.12 | 10.76 | 4.12 | 38.32 | 12.00 | 6.90 | 59.81 |
| **ReS 10** | 684.00 | 0.40 | 499.78 | 645.17 | 0.77 | 145.39 | 0.94 | 0.59 | 62.35 | 52.00 | 7.60 | 7.74 |
| **ReS mean** | 360.20 | 1.69 | 2232.30 | 2422.79 | 0.90 | 190.49 | 7.78 | 4.82 | 67.07 | 32.77 | 8.33 | 60.81 |
| **Max** | 684.00 | 3.58 | 3975.34 | 4201.81 | 0.98 | 421.68 | 13.55 | 6.52 | 97.00 | 65.00 | 11.84 | 106.45 |
| **Min** | 42.00 | 0.18 | 245.88 | 265.51 | 0.77 | 19.63 | 0.94 | 0.59 | 28.37 | 1.70 | 4.05 | 7.74 |
| **Std dev** | 212.14 | 1.27 | 1274.55 | 1310.02 | 0.07 | 121.41 | 3.34 | 1.78 | 23.31 | 22.43 | 2.64 | 24.10 |
| **CV%** | 58.89 | 75.10 | 57.10 | 54.07 | 7.32 | 63.74 | 42.87 | 37.05 | 34.75 | 68.43 | 31.65 | 39.63 |

**Supplementary Table S6.** Correlation matrix of elements and magnetic parameters for all soil types.

| **Forest soils** | **Al** | **B** | **Ba** | **Ca** | **Cr** | **Cu** | **Fe** | **K** | **Mg** | **Mn** | **Ni** | **Pb** | **Zn** | **XLF (10-8m3kg-1)** | **XARM (10-5m3kg-1)** | **IRM300** | **SIRM(10-5Am2kg-1)** | **HIRM (10-5 Am2 kg-1)** | **XFD%** |
| --- | --- | --- | --- | --- | --- | --- | --- | --- | --- | --- | --- | --- | --- | --- | --- | --- | --- | --- | --- |
| **Al** | 1.00 |  |  |  |  |  |  |  |  |  |  |  |  |  |  |  |  |  |  |
| **B** | 0.36 | 1.00 |  |  |  |  |  |  |  |  |  |  |  |  |  |  |  |  |  |
| **Ba** | -0.32 | -0.50 | 1.00 |  |  |  |  |  |  |  |  |  |  |  |  |  |  |  |  |
| **Ca** | -0.23 | -0.24 | 0.35 | 1.00 |  |  |  |  |  |  |  |  |  |  |  |  |  |  |  |
| **Cr** | 0.25 | 0.96 | -0.39 | -0.16 | 1.00 |  |  |  |  |  |  |  |  |  |  |  |  |  |  |
| **Cu** | -0.31 | 0.02 | -0.17 | -0.10 | 0.04 | 1.00 |  |  |  |  |  |  |  |  |  |  |  |  |  |
| **Fe** | 0.34 | 1.00 | -0.52 | -0.20 | 0.95 | 0.04 | 1.00 |  |  |  |  |  |  |  |  |  |  |  |  |
| **K** | -0.19 | -0.50 | 0.93 | 0.04 | -0.42 | -0.18 | -0.54 | 1.00 |  |  |  |  |  |  |  |  |  |  |  |
| **Mg** | -0.44 | -0.52 | 0.77 | 0.42 | -0.46 | -0.10 | -0.53 | 0.63 | 1.00 |  |  |  |  |  |  |  |  |  |  |
| **Mn** | 0.19 | 0.56 | 0.13 | 0.09 | 0.56 | -0.14 | 0.54 | 0.01 | 0.37 | 1.00 |  |  |  |  |  |  |  |  |  |
| **Ni** | 0.41 | 0.95 | -0.50 | -0.13 | 0.96 | 0.03 | 0.94 | -0.54 | -0.46 | 0.62 | 1.00 |  |  |  |  |  |  |  |  |
| **Pb** | -0.32 | 0.00 | -0.19 | -0.08 | 0.01 | 1.00 | 0.02 | -0.20 | -0.08 | -0.14 | 0.01 | 1.00 |  |  |  |  |  |  |  |
| **Zn** | -0.13 | 0.46 | -0.06 | 0.03 | 0.51 | 0.77 | 0.47 | -0.13 | 0.01 | 0.45 | 0.50 | 0.76 | 1.00 |  |  |  |  |  |  |
| **XLF (10-8m3kg-1)** | 0.55 | -0.33 | -0.06 | -0.03 | -0.36 | -0.20 | -0.35 | 0.01 | -0.22 | -0.33 | -0.20 | -0.20 | -0.39 | 1.00 |  |  |  |  |  |
| **XARM (10-5m3kg-1)** | 0.62 | -0.23 | -0.45 | -0.24 | -0.32 | -0.15 | -0.24 | -0.33 | -0.39 | -0.40 | -0.11 | -0.14 | -0.47 | 0.76 | 1.00 |  |  |  |  |
| **IRM300** | 0.43 | -0.31 | -0.01 | 0.06 | -0.30 | -0.20 | -0.33 | 0.01 | -0.23 | -0.35 | -0.18 | -0.20 | -0.37 | 0.97 | 0.64 | 1.00 |  |  |  |
| **SIRM(10-5Am2kg-1)** | 0.43 | -0.31 | -0.02 | 0.06 | -0.30 | -0.18 | -0.33 | 0.01 | -0.23 | -0.36 | -0.18 | -0.18 | -0.37 | 0.97 | 0.64 | 1.00 | 1.00 |  |  |
| **HIRM (10-5 Am2 kg-1)** | -0.14 | -0.04 | -0.20 | 0.17 | -0.02 | 0.66 | 0.00 | -0.27 | -0.43 | -0.55 | -0.07 | 0.64 | 0.31 | 0.04 | 0.08 | 0.10 | 0.12 | 1.00 |  |
| **XFD%** | 0.20 | -0.05 | 0.09 | -0.01 | -0.19 | -0.10 | -0.05 | 0.10 | 0.54 | 0.56 | -0.04 | -0.06 | 0.08 | -0.17 | 0.01 | -0.33 | -0.34 | -0.58 | 1.00 |

| **Road soils** | **Al** | **B** | **Ba** | **Ca** | **Cr** | **Cu** | **Fe** | **K** | **Mg** | **Mn** | **Ni** | **Pb** | **Zn** | **XLF (10-8m3kg-1)** | **XARM (10-5m3kg-1)** | **IRM300** | **SIRM(10-5Am2kg-1)** | **HIRM (10-5 Am2 kg-1)** | **XFD%** |
| --- | --- | --- | --- | --- | --- | --- | --- | --- | --- | --- | --- | --- | --- | --- | --- | --- | --- | --- | --- |
| **Al** | 1.00 |  |  |  |  |  |  |  |  |  |  |  |  |  |  |  |  |  |  |
| **B** | 0.33 | 1.00 |  |  |  |  |  |  |  |  |  |  |  |  |  |  |  |  |  |
| **Ba** | 0.59 | -0.51 | 1.00 |  |  |  |  |  |  |  |  |  |  |  |  |  |  |  |  |
| **Ca** | 0.70 | -0.10 | 0.62 | 1.00 |  |  |  |  |  |  |  |  |  |  |  |  |  |  |  |
| **Cr** | 0.25 | 0.90 | -0.54 | -0.09 | 1.00 |  |  |  |  |  |  |  |  |  |  |  |  |  |  |
| **Cu** | 0.76 | 0.60 | 0.15 | 0.52 | 0.56 | 1.00 |  |  |  |  |  |  |  |  |  |  |  |  |  |
| **Fe** | 0.37 | 0.94 | -0.49 | -0.02 | 0.96 | 0.60 | 1.00 |  |  |  |  |  |  |  |  |  |  |  |  |
| **K** | 0.54 | -0.53 | 0.96 | 0.48 | -0.61 | 0.04 | -0.54 | 1.00 |  |  |  |  |  |  |  |  |  |  |  |
| **Mg** | 0.76 | 0.02 | 0.58 | 0.94 | 0.04 | 0.58 | 0.11 | 0.45 | 1.00 |  |  |  |  |  |  |  |  |  |  |
| **Mn** | 0.59 | 0.53 | 0.02 | 0.61 | 0.57 | 0.69 | 0.67 | -0.15 | 0.64 | 1.00 |  |  |  |  |  |  |  |  |  |
| **Ni** | 0.77 | 0.43 | 0.31 | 0.67 | 0.32 | 0.93 | 0.37 | 0.21 | 0.67 | 0.56 | 1.00 |  |  |  |  |  |  |  |  |
| **Pb** | 0.66 | 0.36 | 0.15 | 0.66 | 0.40 | 0.84 | 0.43 | 0.05 | 0.76 | 0.61 | 0.82 | 1.00 |  |  |  |  |  |  |  |
| **Zn** | 0.75 | 0.45 | 0.28 | 0.68 | 0.37 | 0.94 | 0.41 | 0.15 | 0.69 | 0.62 | 0.99 | 0.84 | 1.00 |  |  |  |  |  |  |
| **XLF (10-8m3kg-1)** | 0.07 | 0.71 | -0.53 | 0.08 | 0.63 | 0.39 | 0.71 | -0.64 | 0.06 | 0.73 | 0.24 | 0.21 | 0.29 | 1.00 |  |  |  |  |  |
| **XARM (10-5m3kg-1)** | 0.51 | 0.63 | -0.12 | 0.54 | 0.63 | 0.63 | 0.63 | -0.25 | 0.60 | 0.65 | 0.66 | 0.68 | 0.70 | 0.50 | 1.00 |  |  |  |  |
| **IRM300** | 0.43 | 0.84 | -0.35 | 0.33 | 0.71 | 0.73 | 0.81 | -0.45 | 0.36 | 0.80 | 0.64 | 0.59 | 0.67 | 0.87 | 0.77 | 1.00 |  |  |  |
| **SIRM(10-5Am2kg-1)** | 0.41 | 0.86 | -0.39 | 0.29 | 0.74 | 0.72 | 0.83 | -0.48 | 0.33 | 0.79 | 0.62 | 0.58 | 0.65 | 0.87 | 0.77 | 1.00 | 1.00 |  |  |
| **HIRM (10-5 Am2 kg-1)** | 0.01 | 0.87 | -0.76 | -0.23 | 0.94 | 0.40 | 0.89 | -0.80 | -0.09 | 0.42 | 0.20 | 0.34 | 0.24 | 0.65 | 0.62 | 0.70 | 0.74 | 1.00 |  |
| **XFD%** | 0.00 | -0.29 | 0.22 | -0.28 | -0.34 | -0.04 | -0.32 | 0.36 | -0.40 | -0.52 | -0.02 | -0.20 | -0.10 | -0.52 | -0.60 | -0.45 | -0.46 | -0.40 | 1.00 |

| **Industrial Soils** | **Al** | **B** | **Ba** | **Ca** | **Cr** | **Cu** | **Fe** | **K** | **Mg** | **Mn** | **Ni** | **Pb** | **Zn** | **XLF (10-8m3kg-1)** | **XARM (10-5m3kg-1)** | **IRM300** | **SIRM(10-5Am2kg-1)** | **HIRM (10-5 Am2 kg-1)** | **XFD%** |
| --- | --- | --- | --- | --- | --- | --- | --- | --- | --- | --- | --- | --- | --- | --- | --- | --- | --- | --- | --- |
| **Al** | 1.00 |  |  |  |  |  |  |  |  |  |  |  |  |  |  |  |  |  |  |
| **B** | 0.38 | 1.00 |  |  |  |  |  |  |  |  |  |  |  |  |  |  |  |  |  |
| **Ba** | 0.70 | -0.11 | 1.00 |  |  |  |  |  |  |  |  |  |  |  |  |  |  |  |  |
| **Ca** | 0.57 | 0.23 | 0.69 | 1.00 |  |  |  |  |  |  |  |  |  |  |  |  |  |  |  |
| **Cr** | 0.17 | 0.52 | -0.32 | -0.32 | 1.00 |  |  |  |  |  |  |  |  |  |  |  |  |  |  |
| **Cu** | 0.14 | 0.26 | -0.01 | 0.40 | -0.01 | 1.00 |  |  |  |  |  |  |  |  |  |  |  |  |  |
| **Fe** | 0.16 | 0.61 | -0.50 | -0.20 | 0.81 | 0.24 | 1.00 |  |  |  |  |  |  |  |  |  |  |  |  |
| **K** | 0.72 | 0.10 | 0.96 | 0.77 | -0.28 | -0.01 | -0.41 | 1.00 |  |  |  |  |  |  |  |  |  |  |  |
| **Mg** | 0.59 | -0.04 | 0.55 | 0.61 | -0.35 | -0.04 | -0.28 | 0.53 | 1.00 |  |  |  |  |  |  |  |  |  |  |
| **Mn** | 0.35 | 0.43 | -0.22 | 0.09 | 0.76 | 0.28 | 0.81 | -0.19 | 0.06 | 1.00 |  |  |  |  |  |  |  |  |  |
| **Ni** | 0.26 | 0.55 | -0.24 | -0.32 | 0.98 | 0.02 | 0.75 | -0.20 | -0.34 | 0.71 | 1.00 |  |  |  |  |  |  |  |  |
| **Pb** | -0.21 | 0.24 | 0.08 | 0.23 | -0.20 | -0.07 | -0.15 | 0.21 | -0.06 | -0.42 | -0.26 | 1.00 |  |  |  |  |  |  |  |
| **Zn** | 0.32 | 0.65 | 0.01 | -0.08 | 0.50 | 0.21 | 0.40 | 0.07 | -0.13 | 0.17 | 0.59 | 0.33 | 1.00 |  |  |  |  |  |  |
| **XLF (10-8m3kg-1)** | 0.14 | 0.55 | -0.38 | -0.28 | **0.94** | 0.17 | **0.84** | -0.33 | -0.38 | **0.79** | **0.94** | -0.18 | 0.59 | 1.00 |  |  |  |  |  |
| **XARM (10-5m3kg-1)** | 0.18 | 0.57 | -0.31 | -0.15 | **0.90** | 0.33 | **0.82** | -0.26 | -0.39 | **0.81** | **0.90** | -0.20 | 0.55 | 0.98 | 1.00 |  |  |  |  |
| **IRM300** | 0.04 | 0.55 | -0.54 | -0.39 | **0.77** | 0.23 | **0.83** | -0.50 | -0.38 | 0.66 | **0.78** | -0.13 | 0.65 | 0.92 | 0.88 | 1.00 |  |  |  |
| **SIRM(10-5Am2kg-1)** | 0.02 | 0.53 | -0.55 | -0.37 | 0.74 | 0.28 | **0.84** | -0.51 | -0.39 | 0.65 | **0.74** | -0.10 | 0.64 | 0.90 | 0.87 | 1.00 | 1.00 |  |  |
| **HIRM (10-5 Am2 kg-1)** | -0.16 | 0.18 | -0.46 | -0.04 | 0.20 | 0.59 | 0.60 | -0.44 | -0.28 | 0.40 | 0.13 | 0.14 | 0.26 | 0.43 | 0.47 | 0.61 | 0.67 | 1.00 |  |
| **XFD%** | 0.72 | 0.08 | **0.76** | 0.71 | -0.10 | 0.18 | -0.07 | 0.78 | 0.47 | 0.10 | -0.09 | -0.06 | -0.13 | -0.24 | -0.14 | -0.45 | -0.45 | -0.25 | 1.00 |

| **Agricultural Soils** | **Al** | **B** | **Ba** | **Ca** | **Cr** | **Cu** | **Fe** | **K** | **Mg** | **Mn** | **Ni** | **Pb** | **Zn** | **XLF (10-8m3kg-1)** | **XARM (10-5m3kg-1)** | **IRM300** | **SIRM(10-5Am2kg-1)** | **HIRM (10-5 Am2 kg-1)** | **XFD%** |
| --- | --- | --- | --- | --- | --- | --- | --- | --- | --- | --- | --- | --- | --- | --- | --- | --- | --- | --- | --- |
| **Al** | 1.00 |  |  |  |  |  |  |  |  |  |  |  |  |  |  |  |  |  |  |
| **B** | 0.08 | 1.00 |  |  |  |  |  |  |  |  |  |  |  |  |  |  |  |  |  |
| **Ba** | -0.16 | -0.47 | 1.00 |  |  |  |  |  |  |  |  |  |  |  |  |  |  |  |  |
| **Ca** | -0.46 | -0.49 | 0.53 | 1.00 |  |  |  |  |  |  |  |  |  |  |  |  |  |  |  |
| **Cr** | -0.46 | 0.62 | -0.08 | -0.27 | 1.00 |  |  |  |  |  |  |  |  |  |  |  |  |  |  |
| **Cu** | 0.71 | 0.39 | -0.37 | -0.20 | -0.22 | 1.00 |  |  |  |  |  |  |  |  |  |  |  |  |  |
| **Fe** | 0.15 | 0.99 | -0.51 | -0.54 | 0.57 | 0.43 | 1.00 |  |  |  |  |  |  |  |  |  |  |  |  |
| **K** | -0.13 | -0.66 | 0.95 | 0.47 | -0.26 | -0.46 | -0.69 | 1.00 |  |  |  |  |  |  |  |  |  |  |  |
| **Mg** | 0.14 | -0.18 | 0.82 | 0.15 | 0.00 | -0.23 | -0.18 | 0.70 | 1.00 |  |  |  |  |  |  |  |  |  |  |
| **Mn** | 0.03 | 0.82 | -0.18 | -0.33 | 0.71 | 0.35 | 0.78 | -0.41 | 0.06 | 1.00 |  |  |  |  |  |  |  |  |  |
| **Ni** | -0.31 | 0.66 | -0.11 | -0.37 | 0.98 | -0.09 | 0.61 | -0.30 | 0.02 | 0.80 | 1.00 |  |  |  |  |  |  |  |  |
| **Pb** | -0.34 | 0.06 | 0.02 | 0.44 | -0.09 | -0.20 | 0.07 | -0.08 | 0.03 | -0.29 | -0.23 | 1.00 |  |  |  |  |  |  |  |
| **Zn** | 0.18 | 0.55 | 0.21 | 0.01 | 0.32 | 0.54 | 0.51 | -0.01 | 0.23 | 0.77 | 0.42 | -0.25 | 1.00 |  |  |  |  |  |  |
| **XLF (10-8m3kg-1)** | -0.01 | -0.07 | 0.45 | 0.26 | 0.22 | -0.21 | -0.06 | 0.32 | 0.54 | -0.04 | 0.15 | 0.34 | -0.06 | 1.00 |  |  |  |  |  |
| **XARM (10-5m3kg-1)** | 0.06 | -0.16 | 0.54 | 0.28 | 0.12 | -0.22 | -0.14 | 0.42 | 0.63 | -0.08 | 0.05 | 0.28 | -0.05 | 0.98 | 1.00 |  |  |  |  |
| **IRM300** | -0.05 | -0.08 | 0.37 | 0.30 | 0.22 | -0.21 | -0.07 | 0.24 | 0.45 | -0.06 | 0.13 | 0.38 | -0.13 | 0.99 | 0.96 | 1.00 |  |  |  |
| **SIRM(10-5Am2kg-1)** | -0.09 | -0.09 | 0.33 | 0.31 | 0.21 | -0.23 | -0.08 | 0.20 | 0.43 | -0.09 | 0.12 | 0.44 | -0.19 | 0.97 | 0.94 | 0.99 | 1.00 |  |  |
| **HIRM (10-5 Am2 kg-1)** | -0.30 | -0.15 | -0.16 | 0.23 | 0.02 | -0.21 | -0.14 | -0.22 | 0.01 | -0.20 | -0.04 | 0.55 | -0.49 | 0.19 | 0.13 | 0.28 | 0.40 | 1.00 |  |
| **XFD%** | 0.31 | 0.00 | 0.39 | 0.04 | -0.07 | 0.00 | 0.02 | 0.28 | 0.72 | 0.29 | -0.01 | -0.12 | 0.27 | 0.35 | 0.47 | 0.31 | 0.29 | -0.01 | 1.00 |

| **Residential soils** | **Al** | **B** | **Ba** | **Ca** | **Cr** | **Cu** | **Fe** | **K** | **Mg** | **Mn** | **Ni** | **Pb** | **Zn** | **XLF (10-8m3kg-1)** | **XAs RM (10-5m3kg-1)** | **IRM300** | **SIRM(10-5Am2kg-1)** | **HIRM (10-5 Am2 kg-1)** | **XFD%** |
| --- | --- | --- | --- | --- | --- | --- | --- | --- | --- | --- | --- | --- | --- | --- | --- | --- | --- | --- | --- |
| **Al** | 1.00 |  |  |  |  |  |  |  |  |  |  |  |  |  |  |  |  |  |  |
| **B** | 0.34 | 1.00 |  |  |  |  |  |  |  |  |  |  |  |  |  |  |  |  |  |
| **Ba** | -0.58 | 0.20 | 1.00 |  |  |  |  |  |  |  |  |  |  |  |  |  |  |  |  |
| **Ca** | -0.48 | 0.16 | 0.85 | 1.00 |  |  |  |  |  |  |  |  |  |  |  |  |  |  |  |
| **Cr** | 0.14 | 0.78 | 0.31 | 0.03 | 1.00 |  |  |  |  |  |  |  |  |  |  |  |  |  |  |
| **Cu** | 0.01 | -0.14 | 0.17 | 0.19 | -0.16 | 1.00 |  |  |  |  |  |  |  |  |  |  |  |  |  |
| **Fe** | 0.32 | 1.00 | 0.21 | 0.14 | 0.81 | -0.13 | 1.00 |  |  |  |  |  |  |  |  |  |  |  |  |
| **K** | -0.64 | 0.10 | 0.97 | 0.82 | 0.24 | 0.06 | 0.11 | 1.00 |  |  |  |  |  |  |  |  |  |  |  |
| **Mg** | -0.43 | 0.09 | 0.88 | 0.86 | 0.01 | 0.35 | 0.08 | 0.85 | 1.00 |  |  |  |  |  |  |  |  |  |  |
| **Mn** | -0.17 | 0.38 | 0.58 | 0.37 | 0.32 | 0.51 | 0.40 | 0.49 | 0.66 | 1.00 |  |  |  |  |  |  |  |  |  |
| **Ni** | 0.51 | 0.57 | -0.04 | -0.23 | 0.66 | 0.34 | 0.60 | -0.09 | -0.06 | 0.50 | 1.00 |  |  |  |  |  |  |  |  |
| **Pb** | -0.34 | 0.04 | 0.68 | 0.95 | -0.16 | 0.22 | 0.02 | 0.64 | 0.80 | 0.29 | -0.29 | 1.00 |  |  |  |  |  |  |  |
| **Zn** | -0.21 | 0.26 | 0.84 | 0.84 | 0.25 | 0.36 | 0.25 | 0.74 | 0.90 | 0.60 | 0.11 | 0.81 | 1.00 |  |  |  |  |  |  |
| **XLF (10-8m3kg-1)** | 0.27 | 0.11 | -0.13 | -0.33 | 0.22 | -0.01 | 0.11 | -0.29 | -0.25 | 0.11 | 0.11 | -0.27 | -0.03 | 1.00 |  |  |  |  |  |
| **XARM (10-5m3kg-1)** | 0.24 | 0.43 | 0.23 | -0.17 | 0.57 | 0.29 | 0.46 | 0.12 | 0.15 | 0.72 | 0.71 | -0.26 | 0.25 | 0.59 | 1.00 |  |  |  |  |
| **IRM300** | -0.08 | 0.27 | 0.56 | 0.15 | 0.57 | 0.35 | 0.31 | 0.48 | 0.40 | 0.74 | 0.58 | 0.02 | 0.48 | 0.44 | 0.89 | 1.00 |  |  |  |
| **SIRM(10-5Am2kg-1)** | -0.10 | 0.31 | 0.60 | 0.21 | 0.60 | 0.34 | 0.35 | 0.51 | 0.42 | 0.74 | 0.58 | 0.08 | 0.53 | 0.43 | 0.87 | 1.00 | 1.00 |  |  |
| **HIRM (10-5 Am2 kg-1)** | -0.15 | 0.54 | 0.60 | 0.72 | 0.52 | -0.01 | 0.55 | 0.52 | 0.42 | 0.23 | 0.15 | 0.67 | 0.64 | -0.03 | 0.02 | 0.25 | 0.33 | 1.00 |  |
| **XFD%** | 0.46 | 0.66 | -0.09 | -0.29 | 0.55 | 0.22 | 0.68 | -0.24 | -0.20 | 0.43 | 0.66 | -0.38 | -0.07 | 0.55 | 0.76 | 0.52 | 0.51 | 0.08 | 1.00 |

**Supplementary Table S7**. Average, Maximum, Minimum, Standard deviation, and Coefficient Variable % of Elemental Concentrations (mg/kg) of different land types in our study area.

| **Sample ID** | **Al** | **B** | **Ba** | **Ca** | **Cr** | **Cu** | **Fe** | **K** | **Mg** | **Mn** | **Ni** | **Pb** | **Zn** |
| --- | --- | --- | --- | --- | --- | --- | --- | --- | --- | --- | --- | --- | --- |
| **FS1** | 73085.52 | 116.85 | 149.61 | 3694.04 | 1028.67 | 36.18 | 72446.36 | 5852.62 | 3483.75 | 942.62 | 105.97 | 21.15 | 103.42 |
| **FS2** | 65975.40 | 87.58 | 74.79 | 2315.71 | 323.89 | 20.91 | 57016.83 | 2890.83 | 2293.72 | 299.00 | 57.56 | 14.74 | 66.56 |
| **FS3** | 73171.10 | 136.33 | 59.54 | 1939.61 | 759.22 | 37.41 | 83916.55 | 2936.37 | 1457.52 | 825.47 | 81.94 | ND | 81.47 |
| **FS4** | 58141.32 | 172.17 | 78.53 | 2014.48 | 906.87 | ND | 103873.05 | 3072.33 | 2055.59 | 549.48 | 79.38 | 26.72 | 85.33 |
| **FS5** | 48388.58 | 208.46 | 91.50 | 1280.34 | 4919.53 | ND | 118688.25 | 2751.39 | 2484.21 | 2788.45 | 552.88 | ND | 112.31 |
| **FS6** | 85056.07 | 76.36 | 79.01 | 1465.74 | 247.09 | ND | 52627.67 | 3824.65 | 2251.49 | 262.29 | 61.49 | ND | 65.53 |
| **FS7** | 94008.57 | 185.39 | 89.78 | 1818.58 | 524.06 | 84.54 | 111364.42 | 2914.19 | 2898.22 | 2468.10 | 133.13 | ND | 130.86 |
| **FS8** | 53101.61 | 39.27 | 160.46 | 3586.79 | 76.36 | ND | 23013.38 | 8117.42 | 2588.33 | 226.11 | 31.27 | ND | 101.63 |
| **FS9** | 107657.47 | 162.24 | 65.07 | 1042.01 | 540.92 | 114.70 | 100659.10 | 2149.57 | 1830.81 | 1177.44 | 135.73 | ND | 111.27 |
| **FS10** | 91559.13 | 102.92 | 158.20 | 1144.36 | 654.09 | ND | 66581.22 | 7896.17 | 4229.45 | 637.78 | 109.00 | ND | 90.36 |
| **FS mean** | 75014.48 | 128.76 | 100.65 | 2030.17 | 998.07 | 58.75 | 79018.68 | 4240.55 | 2557.31 | 1017.67 | 134.84 | 20.87 | 94.87 |
| **Max** | 107657.47 | 208.46 | 160.46 | 3694.04 | 4919.53 | 114.70 | 118688.25 | 8117.42 | 4229.45 | 2788.45 | 552.88 | 26.72 | 130.86 |
| **Min** | 48388.58 | 39.27 | 59.54 | 1042.01 | 76.36 | 20.91 | 23013.38 | 2149.57 | 1457.52 | 226.11 | 31.27 | 14.74 | 65.53 |
| **Std dev** | 19332.01 | 53.63 | 39.54 | 940.93 | 1408.87 | 39.34 | 30267.34 | 2221.15 | 811.65 | 905.82 | 150.63 | 6.00 | 20.92 |
| **CV%** | 25.77 | 41.65 | 39.28 | 46.35 | 141.16 | 66.97 | 38.30 | 52.38 | 31.74 | 89.01 | 111.72 | 28.72 | 22.05 |
|  | | | | | | | | | | | | | |
| **Sample ID** | **Al** | **B** | **Ba** | **Ca** | **Cr** | **Cu** | **Fe** | **K** | **Mg** | **Mn** | **Ni** | **Pb** | **Zn** |
| **RoS 1** | 46013.17 | 79.21 | 179.16 | 9310.97 | 188.55 | ND | 32247.40 | 8747.78 | 1476.00 | 222.53 | ND | 0.00 | 98.92 |
| **RoS 2** | 74281.11 | 96.27 | 284.13 | 17407.78 | 324.70 | 137.34 | 48063.52 | 12752.14 | 4480.00 | 346.30 | 42.18 | 110.31 | 495.14 |
| **RoS 3** | 53801.30 | 96.52 | 176.66 | 9491.83 | 418.02 | 27.11 | 51603.91 | 8152.84 | 1982.81 | 250.39 | ND | 32.46 | 118.76 |
| **RoS 4** | 59720.90 | 100.45 | 212.08 | 11288.48 | 353.25 | 36.97 | 53414.28 | 9671.11 | 2047.18 | 334.39 | ND | ND | 115.91 |
| **RoS 5** | 51814.08 | 61.60 | 230.06 | 11567.82 | 245.05 | 34.56 | 33628.61 | 9977.01 | 2529.34 | 273.03 | ND | 48.52 | 126.21 |
| **RoS 6** | 62511.01 | 43.35 | 339.23 | 13507.08 | 108.19 | ND | 21287.48 | 16437.28 | 3223.57 | 234.91 | ND | ND | 104.15 |
| **RoS 7** | 54152.10 | 46.86 | 261.23 | 9514.37 | 126.27 | 11.89 | 24410.76 | 13407.87 | 1224.72 | 186.10 | ND | ND | 83.23 |
| **RoS 8** | 46778.49 | 41.20 | 244.17 | 13394.15 | 167.31 | ND | 23982.70 | 10258.55 | 2328.25 | 262.60 | ND | ND | 131.21 |
| **RoS 9** | 54053.87 | 59.26 | 234.09 | 11752.87 | 229.85 | 25.86 | 36575.21 | 11074.09 | 2773.36 | 289.90 | ND | 33.50 | 115.60 |
| **RoS 10** | 58348.91 | 47.41 | 252.07 | 15476.72 | 149.66 | ND | 30206.49 | 12324.66 | 3627.36 | 279.54 | ND | 44.63 | 100.83 |
| **RoS mean** | 56147.49 | 67.21 | 241.29 | 12271.21 | 231.08 | 45.62 | 35542.04 | 11280.33 | 2569.26 | 267.97 | 42.18 | 44.91 | 149.00 |
| **Max** | 74281.11 | 100.45 | 339.23 | 17407.78 | 418.02 | 137.34 | 53414.28 | 16437.28 | 4480.00 | 346.30 | 42.18 | 110.31 | 495.14 |
| **Min** | 46013.17 | 41.20 | 176.66 | 9310.97 | 108.19 | 11.89 | 21287.48 | 8152.84 | 1224.72 | 186.10 | 42.18 | 0.00 | 83.23 |
| **Std dev** | 8233.19 | 23.80 | 48.33 | 2692.90 | 104.04 | 45.78 | 11732.93 | 2493.26 | 995.51 | 48.75 | #DIV/0! | 36.31 | 122.45 |
| **CV%** | 14.66 | 35.41 | 20.03 | 21.94 | 45.02 | 100.35 | 33.01 | 22.10 | 38.75 | 18.19 | #DIV/0! | 80.86 | 82.18 |
|  | | | | | | | | | | | | | |
| **Sample ID** | **Al** | **B** | **Ba** | **Ca** | **Cr** | **Cu** | **Fe** | **K** | **Mg** | **Mn** | **Ni** | **Pb** | **Zn** |
| **AS1** | 74877.23 | 195.46 | 131.74 | 1445.25 | 377.22 | 49.52 | 50231.45 | 8064.09 | 1940.62 | 143.90 | 67.77 | 28.09 | 191.09 |
| **AS2** | 77609.11 | 172.93 | 79.92 | 714.23 | 568.34 | 48.27 | 62676.09 | 3701.32 | 1693.14 | 249.75 | 121.38 | ND | 207.03 |
| **AS3** | 103329.77 | 123.89 | 207.14 | 1345.22 | 296.68 | 39.61 | 45300.92 | 10426.85 | 2577.95 | 284.51 | 81.08 | ND | 190.91 |
| **AS4** | 87346.78 | 138.29 | 154.18 | 1388.33 | 402.06 | 32.41 | 62220.51 | 8424.42 | 1784.84 | 559.63 | 76.17 | ND | 162.50 |
| **AS5** | 78360.18 | 153.51 | 100.48 | 1389.15 | 522.90 | 263.99 | 74924.99 | 4516.46 | 1853.47 | 743.15 | 87.79 | ND | 186.08 |
| **AS6** | 68600.69 | 130.30 | 102.14 | 918.56 | 471.25 | 44.73 | 67008.41 | 4844.46 | 1586.19 | 166.47 | 67.74 | 29.25 | 203.37 |
| **AS7** | 83118.56 | 189.59 | 92.74 | 767.43 | 2696.41 | 54.53 | 99805.42 | 4394.96 | 1323.75 | 1055.44 | 310.86 | ND | 214.27 |
| **AS8** | 64397.04 | 128.53 | 59.67 | 613.20 | 601.87 | 35.28 | 71328.73 | 2410.48 | 1309.71 | 186.20 | 87.37 | ND | 167.30 |
| **AS9** | 76364.84 | 149.37 | 61.86 | 989.16 | 1035.28 | ND | 84655.46 | 2654.22 | 2342.83 | 924.86 | 113.29 | ND | 170.65 |
| **AS10** | 55666.53 | 57.12 | 105.56 | 757.13 | 115.12 | ND | 28369.67 | 3779.56 | 1651.20 | 80.44 | 41.55 | ND | 158.54 |
| **AS mean** | 76967.07 | 143.90 | 109.54 | 1032.77 | 708.71 | 71.04 | 64652.17 | 5321.68 | 1806.37 | 439.44 | 105.50 | 28.67 | 185.17 |
| **Max** | 103329.77 | 195.46 | 207.14 | 1445.25 | 2696.41 | 263.99 | 99805.42 | 10426.85 | 2577.95 | 1055.44 | 310.86 | 29.25 | 214.27 |
| **Min** | 55666.53 | 57.12 | 59.67 | 613.20 | 115.12 | 32.41 | 28369.67 | 2410.48 | 1309.71 | 80.44 | 41.55 | 28.09 | 158.54 |
| **Std dev** | 13035.72 | 39.57 | 44.83 | 326.60 | 738.29 | 78.32 | 20276.44 | 2698.55 | 403.98 | 355.37 | 75.66 | 0.82 | 19.66 |
| **CV%** | 16.94 | 27.50 | 40.92 | 31.62 | 104.17 | 110.24 | 31.36 | 50.71 | 22.36 | 80.87 | 71.72 | 2.87 | 10.62 |
|  | | | | | | | | | | | | | |
| **Sample ID** | **Al** | **B** | **Ba** | **Ca** | **Cr** | **Cu** | **Fe** | **K** | **Mg** | **Mn** | **Ni** | **Pb** | **Zn** |
| **IS 1** | 89997.69 | 241.73 | 74.70 | 956.43 | 2027.95 | 84.86 | 118967.86 | 2844.70 | 1115.42 | 1066.26 | 320.00 | 27.31 | 204.52 |
| **IS 2** | 71418.59 | 130.82 | 123.75 | 4778.22 | 603.92 | ND | 74424.92 | 5199.07 | 1566.61 | 273.15 | 42.57 | ND | 86.09 |
| **IS 3** | 51160.09 | 85.34 | 162.76 | 5280.98 | 477.00 | 717.49 | 52856.33 | 6102.57 | 2734.83 | 262.61 | 42.06 | 331.06 | 246.21 |
| **IS 4** | 64696.82 | 75.58 | 347.98 | 24540.87 | 547.90 | 26.79 | 47965.74 | 10424.97 | 3649.90 | 363.58 | 39.14 | ND | 116.44 |
| **IS 5** | 59017.14 | 71.27 | 260.83 | 12200.55 | 425.83 | ND | 44455.76 | 8810.03 | 2999.40 | 328.76 | 27.05 | ND | 97.38 |
| **IS 6** | 52205.54 | 71.20 | 176.10 | 10259.57 | 428.95 | ND | 45200.92 | 7499.68 | 3475.20 | 346.72 | 34.85 | ND | 98.36 |
| **IS 7** | 100584.26 | 63.40 | 59.53 | 1586.28 | 108.30 | ND | 40916.83 | 3594.09 | 1392.52 | 114.49 | 34.95 | ND | 47.00 |
| **IS 8** | 47664.93 | 68.85 | 303.40 | ND | 328.30 | ND | 41872.73 | 10962.98 | 5042.39 | 645.32 | ND | ND | 91.30 |
| **IS 9** | 84354.88 | 68.91 | 405.03 | ND | 299.17 | 30.77 | 40852.69 | 18229.69 | 4003.26 | 530.11 | ND | ND | 126.43 |
| **IS 10** | 76618.77 | 89.83 | 274.90 | 20386.99 | 371.56 | 53.58 | 54580.10 | 7938.50 | 5744.16 | 1093.86 | 72.66 | 33.92 | 162.44 |
| **IS mean** | 69771.87 | 96.69 | 218.90 | 9998.74 | 561.89 | 182.70 | 56209.39 | 8160.63 | 3172.37 | 502.48 | 76.66 | 130.77 | 127.62 |
| **Max** | 100584.26 | 241.73 | 405.03 | 24540.87 | 2027.95 | 717.49 | 118967.86 | 18229.69 | 5744.16 | 1093.86 | 320.00 | 331.06 | 246.21 |
| **Min** | 47664.93 | 63.40 | 59.53 | 956.43 | 108.30 | 26.79 | 40852.69 | 2844.70 | 1115.42 | 114.49 | 27.05 | 27.31 | 47.00 |
| **Std dev** | 17968.66 | 54.54 | 117.17 | 8671.37 | 533.36 | 299.85 | 24236.91 | 4439.63 | 1539.12 | 337.17 | 99.24 | 173.49 | 60.21 |
| **CV%** | 25.75 | 56.41 | 53.53 | 86.72 | 94.92 | 164.12 | 43.12 | 54.40 | 48.52 | 67.10 | 129.46 | 132.67 | 47.18 |
|  | | | | | | | | | | | | | |
| **Sample ID** | **Al** | **B** | **Ba** | **Ca** | **Cr** | **Cu** | **Fe** | **K** | **Mg** | **Mn** | **Ni** | **Pb** | **Zn** |
| **ReS 1** | 71430.42 | 103.66 | 232.10 | 10191.95 | 351.49 | 34.60 | 78377.86 | 8774.52 | 4238.31 | 506.67 | 59.73 | 30.42 | 204.27 |
| **ReS 2** | 65131.56 | 84.98 | 140.13 | 3049.57 | 624.79 | ND | 69595.60 | 6629.36 | 1776.65 | 346.83 | 80.21 | ND | 86.77 |
| **ReS 3** | 97131.94 | 173.02 | 111.87 | 3413.99 | 728.88 | ND | 131190.68 | 5002.31 | 1789.61 | 406.75 | 90.62 | ND | 86.35 |
| **ReS 4** | 74733.99 | 111.06 | 173.80 | 3342.09 | 672.79 | 34.47 | 86099.47 | 6483.74 | 2857.35 | 554.61 | 84.58 | ND | 144.10 |
| **ReS 5** | 91549.24 | 90.84 | 102.07 | 1880.04 | 269.99 | 48.43 | 71197.18 | 4686.35 | 2384.65 | 619.00 | 93.19 | ND | 94.65 |
| **ReS 6** | 113660.72 | 126.69 | 148.03 | 2742.00 | 712.05 | ND | 97324.40 | 6309.98 | 2395.99 | 421.64 | 98.05 | ND | 131.81 |
| **ReS 7** | 115601.64 | 100.60 | 70.75 | 1931.68 | 505.26 | 57.80 | 78491.29 | 3544.82 | 1626.62 | 337.24 | 109.88 | ND | 105.42 |
| **ReS 8** | 96298.74 | 74.18 | 68.21 | 1728.81 | 201.81 | ND | 55790.80 | 4516.06 | 2123.17 | 293.56 | 67.23 | ND | 81.50 |
| **ReS 9** | 69828.61 | 57.19 | 142.39 | 3609.16 | 212.51 | 26.28 | 45253.41 | 6611.18 | 2348.13 | 294.28 | 51.92 | ND | 81.33 |
| **ReS 10** | 96244.65 | 73.96 | 45.41 | 1140.01 | 217.29 | ND | 55872.97 | 2627.25 | 978.36 | 147.28 | 42.24 | ND | 63.86 |
| **ReS mean** | 89161.15 | 99.62 | 123.48 | 3302.93 | 449.69 | 40.32 | 76919.37 | 5518.56 | 2251.88 | 392.79 | 77.76 | 30.42 | 108.01 |
| **Max** | 115601.64 | 173.02 | 232.10 | 10191.95 | 728.88 | 57.80 | 131190.68 | 8774.52 | 4238.31 | 619.00 | 109.88 | 30.42 | 204.27 |
| **Min** | 65131.56 | 57.19 | 45.41 | 1140.01 | 201.81 | 26.28 | 45253.41 | 2627.25 | 978.36 | 147.28 | 42.24 | 30.42 | 63.86 |
| **Std dev** | 18084.30 | 32.77 | 55.87 | 2560.51 | 222.10 | 12.60 | 24560.80 | 1789.05 | 871.08 | 139.99 | 21.77 | ND | 41.64 |
| **CV%** | 20.28 | 32.90 | 45.25 | 77.52 | 49.39 | 31.26 | 31.93 | 32.42 | 38.68 | 35.64 | 27.99 | ND | 38.55 |

**Supplementary Table S8.** Principal Component Analysis (PCA) loading matrix showing the correlation coefficients of physico-chemical, environmental magnetic, and elemental variables with the first six principal components (PC1–PC6).

|  | PC 1 | PC 2 | PC 3 | PC 4 | PC 5 | PC 6 |
| --- | --- | --- | --- | --- | --- | --- |
| pH | -0.197 | 0.179 | 0.221 | -0.094 | 0.268 | -0.099 |
| Conductivity | -0.191 | 0.151 | 0.309 | 0.009 | 0.178 | 0.054 |
| Salinity | -0.202 | 0.124 | 0.318 | 0.016 | 0.175 | 0.024 |
| χ_lf_ | 0.336 | 0.128 | 0.089 | 0.077 | 0.129 | -0.001 |
| χ_ARM_ | 0.316 | 0.055 | 0.237 | -0.045 | -0.104 | -0.028 |
| IRM_300mT_ | 0.300 | 0.146 | 0.126 | 0.147 | 0.137 | 0.066 |
| SIRM | 0.293 | 0.150 | 0.137 | 0.157 | 0.137 | 0.063 |
| S-ratio | 0.201 | 0.072 | -0.102 | 0.059 | 0.246 | 0.012 |
| HIRM | -0.110 | 0.096 | 0.271 | 0.247 | 0.019 | -0.069 |
| SIRM/χ_lf_ | -0.162 | -0.045 | 0.289 | 0.135 | -0.123 | 0.355 |
| χ_ARM_/χ_lf_ | 0.308 | 0.000 | 0.242 | -0.034 | -0.148 | -0.022 |
| χ_ARM_/SIRM | 0.329 | 0.011 | 0.185 | -0.062 | -0.116 | -0.071 |
| χ_fd_ | 0.366 | 0.024 | 0.036 | 0.043 | 0.048 | 0.033 |
| χ_fd_ % | 0.191 | -0.202 | -0.206 | 0.015 | -0.120 | 0.065 |
| χ_ARM_/χ_FD_ | -0.028 | 0.129 | 0.283 | -0.087 | 0.205 | -0.103 |
| Al | 0.103 | -0.164 | 0.102 | 0.003 | -0.079 | 0.596 |
| B | -0.030 | -0.342 | 0.103 | 0.108 | 0.100 | 0.187 |
| Ba | 0.007 | 0.288 | -0.271 | 0.096 | 0.153 | 0.212 |
| Ca | -0.002 | 0.270 | -0.133 | 0.148 | 0.250 | -0.053 |
| Cr | 0.034 | -0.282 | -0.018 | 0.081 | 0.366 | -0.160 |
| Cu | -0.029 | -0.045 | -0.005 | 0.529 | -0.239 | -0.208 |
| Fe | 0.024 | -0.352 | 0.055 | 0.090 | 0.192 | -0.012 |
| K | -0.023 | 0.289 | -0.218 | 0.041 | 0.096 | 0.252 |
| Mg | 0.056 | 0.153 | -0.304 | 0.151 | 0.159 | 0.180 |
| Mn | 0.061 | -0.255 | -0.096 | 0.067 | 0.388 | -0.063 |
| Ni | 0.033 | -0.331 | 0.017 | 0.076 | 0.295 | -0.033 |
| Pb | -0.024 | 0.054 | -0.029 | 0.543 | -0.168 | -0.306 |
| Zn | -0.137 | -0.049 | 0.076 | 0.415 | 0.000 | 0.351 |
| ***Eigenvalue*** | ***6.55*** | ***6.17*** | ***3.56*** | ***2.34*** | ***2.11*** | ***1.29*** |
| ***% variance*** | ***23.39*** | ***22.04*** | ***12.71*** | ***8.37*** | ***7.54*** | ***4.62*** |
